# Multi-omics analysis delineates molecular signatures of spinal ependymal tumor

**DOI:** 10.1007/s13402-025-01122-0

**Published:** 2025-10-29

**Authors:** Weihao Liu, Chao Ning, Xiaohan Geng, Bo Wang, Yaowu Zhang, Chong Wang, Yixiang Liu, Guanghao Zheng, Yongzhi Wang, Xinyu Wang, Dong Li, Wenqing Jia

**Affiliations:** 1 Department of Neurosurgery, Beijing Tiantan Hospital, Beijing Neurosurgical Institute, Capital Medical University, Beijing, China; 2 Department of Neurosurgery, Beijing Friendship Hospital, Capital Medical University, Beijing, China; 3 National Laboratory of Biomacromolecules, CAS Center for Excellence in Biomacromolecules, Institute of Biophysics, Chinese Academy of Sciences, Beijing, China; 4 College of Life Sciences, University of Chinese Academy of Sciences, Beijing, China; 5 The State Key Laboratory of Membrane Biology, Peking University Joint Center for Life Sciences, School of Life Sciences, Tsinghua University, Beijing, 100084 China

**Keywords:** Spinal, Ependymoma, Myxopapillary ependymoma, Subependymoma, Multi-omics

## Abstract

**Background:**

Spinal ependymal tumors are a diverse group of neoplasms encompassing four subtypes: spinal ependymoma (SP-EPN), spinal ependymoma, *MYCN*-amplification (SP-EPN-*MYCN*), spinal myxopapillary ependymoma (SP-MPE), and spinal subependymoma (SP-SE). However, the molecular differences among these subtypes remain largely unknown.

**Methods:**

Using an integrated multi-omics approach (whole-genome sequencing, RNA-seq, and mass spectrometry), we identified the distinct molecular characteristics of three subtypes except for SP-EPN-*MYCN*.

**Results:**

In SP-EPN, abnormal enrichment of ciliary signaling, particularly involving the *MKS* complex, was evident. SP-MPE exhibited significant dysregulation of mitochondrial metabolism, reflecting a metabolic profile aligned with the Warburg effect. SP-SE tumors showed enhanced activity of immune-related pathways, including interferon signaling and extracellular vesicle dynamics, suggesting a distinct tumor microenvironment.

**Conclusion:**

This pilot study identifies candidate molecular markers in a single-center spinal ependymal tumor cohort.

**Clinical trial number:**

Not applicable.

**Supplementary Information:**

The online version contains supplementary material available at 10.1007/s13402-025-01122-0.

## Introduction

Spinal ependymal tumors constitute 18.3% of all primary spinal tumors in adult patients and represent 3–6% of all central nervous system (CNS) malignancies [1]. Although surgical resection is the primary treatment, many patients suffer post-operative neurological impairments that affect sensory and motor functions [2]. Additionally, 50–70% of patients who undergo subtotal resection experience local recurrence or distant metastasis [3].

Current clinical classifications divide these tumors into three subtypes: SP-EPN, SP-EPN-*MYCN*, SP-MPE, and SP-SE primarily based on histopathological and molecular pathological features [4]. However, such classifications fail to capture the molecular diversity of these tumors [5]. This underscores the need for molecular profiling to refine tumor classification and improve therapeutic strategies.

To establish a molecular classification, a pioneer study profiled DNA methylation across 500 central nervous system (CNS) ependymal tumors, encompassing all anatomical compartments of the CNS, including the spine, posterior fossa, and supratentorial regions [6]. Nine distinct molecular subgroups were identified in this study. Among these, specific genetic alterations were characteristic of spinal ependymal tumor subgroups: 6q deletions were associated with SP-SE, chromosomal instability was recurrent in SP-MPE, and *NF2* mutations were observed in SP-EPN [6]. Neyazi et al. refined the classification of SP-EPN by integrating transcriptomic, DNA methylation, and clinical data, and identified two molecular subtypes based on the presence or absence of *NF2* mutations [7]. Further investigations have identified *MYCN* amplification as a novel molecular subgroup of SP-EPN [8, 9]. Bockmayr et al. elucidated SP-MPE’s morphological and clinical heterogeneity by distinguishing between two molecularly distinct subtypes [10]. However, the above findings heavily rely on the DNA methylation profiling for spinal ependymal tumor classification. Comprehensive studies integrating WGS, transcriptomics, and proteomics across all subtypes remain limited.

To deepen our understanding of the molecular patterns of different spinal ependymal tumor subtypes, we performed a multi-omics study (WGS, RNA-seq, and mass spectrometry) on 25 spinal ependymal tumors spanning three subtypes. This comprehensive approach reveals preliminary molecular features, providing a valuable resource for the rare cancer research community.

## Materials and methods

### Clinical sample collection

All 25 patients provided informed consent for the use of their tumor tissues in this study. Tumor samples were collected during routine clinical treatment. Fresh tumor tissues from 25 patients were snap-frozen in liquid nitrogen within 5 min of surgical resection. Relevant clinical data were extracted from patients’ electronic medical records.

### Histology and immunohistochemistry

Two neuropathologists independently evaluated the tumor samples according to H&E-staining, immunohistochemistry and categorized them into subtypes: SP-EPN, SP-MPE, SP-SE, or SP-EPN-*MYCN*. The hospital pathology department performed immunohistochemical analysis of FFPE samples during hospitalization. The primary antibodies used for immunohistochemical analysis were GFAP, Ki-67, EMA, H3K27me3, Olig-2, S100, and SOX10. Staining was performed using the SuperPicture™ 3rd Gen Immunohistochemistry Detection Kit.

### WGS library construction

Genomic DNA was extracted from 10 mg of fresh-frozen tumor tissue using a DNeasy Blood & Tissue Kit (Qiagen, Cat. No. 69504) according to the manufacturer’s instructions. WGS was performed on the Illumina NovaSeq 6000 platform (2 × 150 bp). The average sequencing depth was ~ 60× for tumor samples. Reads were aligned to the GRCh38 reference genome using BWA-MEM. PCR duplicates were marked using Picard, and base quality recalibration was done using GATK.

Putative somatic SNVs were filtered against public germline variant databases (including NyuWa Genome resource, gnomAD, dbSNP, and 1000 Genomes) to systematically exclude potential germline polymorphisms or inherited variants. Retention of only variants that were absent in the germline sample, with thresholds of VAF ≥ 0.05, DP ≥ 10, and QUAL ≥ 30. To ensure the validity of these findings, we have taken the following steps: Database filtering: Variants with minor allele frequency (MAF > 0.01) in NyuWa Genome, gnomAD, dbSNP, or 1000 Genomes were excluded. Independent tool confirmation: Variants were confirmed with both Mutect2 and Strelka2, and with manual curation in IGV. Reads were aligned to the GRCh38 reference genome using BWA-MEM (v0.7.17), and duplicates were marked using Picard (v2.23.8). Somatic SNVs and small indels were called using GATK Mutect2 (v4.1.9.0) in tumor–normal pair mode. Variants were filtered by FilterMutectCalls, and background artifacts were excluded.

### RNA-Seq library construction and fusion transcript detection

Total RNA was isolated from 2 mg of frozen tumor tissues using Trizol reagent (Thermo Fisher Scientific, Cat. No. 15596018). RNA-seq libraries were constructed using the NEB Next Ultra™ RNA Library Prep Kit (NEB, Cat. No. E7490) and sequenced using the NovaSeq 6000 System platform. Fusion transcript detection was performed using the Arriba algorithm [11]. 

Clustering analysis was performed on 25 samples using the k-means method in Python. For each gene, the normalized expression was calculated by dividing the expression levels from all samples with its maximum observed transcripts per million (TPM). Hierarchical clustering (HCL) and principal component analysis (PCA) were performed using the kernel PCA method in Python with default settings. Statistically significant GO terms were defined using FDR < 0.05. The KEGG pathway analysis was performed by KofamKOALA [12], and enrichment analysis was carried out using R package clusterProfiler [13].

### Mass spectrometry (MS-spec)

Approximately 10 mg of each tumor sample was homogenized in 500 µL of lysis buffer (100 mM TEAB pH 8.0, 1% SDS, supplemented with 1× protease inhibitor cocktail) using a Precellys Evolution tissue homogenizer with dry ice. Lysates were centrifuged at 20,000 × g for 30 min at 4 °C and the supernatants were collected. Protein concentrations were measured using a BCA assay. For downstream MS-spec analysis, 100 µg of protein was reduced with 5 mM TCEP at 55 °C for 1 h and alkylated with 10 mM IAA at room temperature for 30 min in the dark. Proteins were precipitated using a standard methanol/chloroform protocol, followed by digestion with 4 µg sequencing-grade modified trypsin (Promega, Cat. No. V5117) at 37 °C for 12 h. The resulting peptides were acidified with TFA to pH ~ 3, desalted using C18 Zip-Tips, and dried using Speed-Vac. The desalted peptides were subjected to LC-MS/MS analysis using an Orbitrap Exploris™ 480 mass spectrometer (Thermo Scientific) coupled to a Proxeon Easy-nLC 1200 system (Thermo Scientific). Data were processed using the SEQUEST HT search engine in the Thermo Proteome Discoverer software (v2.4.1.15) against the UniProt human reference proteome database.

### Immunofluorescence staining of FFPE samples

The FFPE sample slides were deparaffinized and rehydrated by sequential immersion in BioDewax and Clear Solution (Servicebio), 50% BioDewax and Clear Solution mixed with 50% ethanol, 100% ethanol, 95% ethanol, 70% ethanol, and 50% ethanol for 10 min each, and deionized water for 30 min. Antigen retrieval was performed by heating slides in 10 mM sodium citrate buffer (pH 6.0) at 95 °C for 10 min. The slides were washed twice with permeabilization buffer (1% donkey serum and 0.4% Triton X-100 in PBS) and blocked with 5% donkey serum in PBS for 30 min at room temperature. Primary antibodies diluted 200 folds in 1% donkey serum in PBS were incubated with slides for 1–2 h at room temperature. Primary antibodies used were rabbit anti-*AQP4* (Proteintech, 16473-1-AP), rabbit anti-GFAP (Proteintech, 16825-1-AP), rabbit anti-*TCTN1* (Proteintech, 15004-1-AP), rabbit anti-*MKS1* (Proteintech, 16206-1-AP), and rabbit anti-*ISG15* (Proteintech, 15981-1-AP). Alexa Fluor 488-conjugated secondary antibodies (Life Technologies) were added to the slides at a dilution of 1/1000 and incubated for 1 h at room temperature.

### Targeted gene panel sequencing

Targeted gene panel sequencing was performed on FFPE samples from 8 out of 25 patients. In brief, this panel-seq analyzed 86 genes for point mutations, insertions, and deletions; 28 genes for copy number variations; 44 gene exons; and 88 rearrangement events, focusing on the genomic alternations relevant to brain tumors. Detailed results are provided in the Supplementary Materials section.

### Data availability

Raw WGS and RNA-seq data were deposited in the China National Center for Bioinformation (https://ngdc.cncb.ac.cn/gsa-human/) under the accession number HRA008375. The mass spectrometry proteomics data have been deposited to the ProteomeXchange Consortium (https://proteomecentral.proteomexchange.org) via the iProX partner repository with the dataset identifier PXD056112.

### Statistical

Continuous variables were analyzed using non-parametric *Wilcoxon* rank-sum tests (two-group comparisons) or Kruskal-Wallis tests (multi-group comparisons), with Benjamini-Hochberg correction for multiple hypothesis testing (FDR < 0.05). Categorical variables were assessed through Fisher’s exact tests. All statistical analyses were performed using R v4.2.2.

## RESULTS

### The basic information of the spinal ependymal tumor’s cohort

**Fig. 1:**
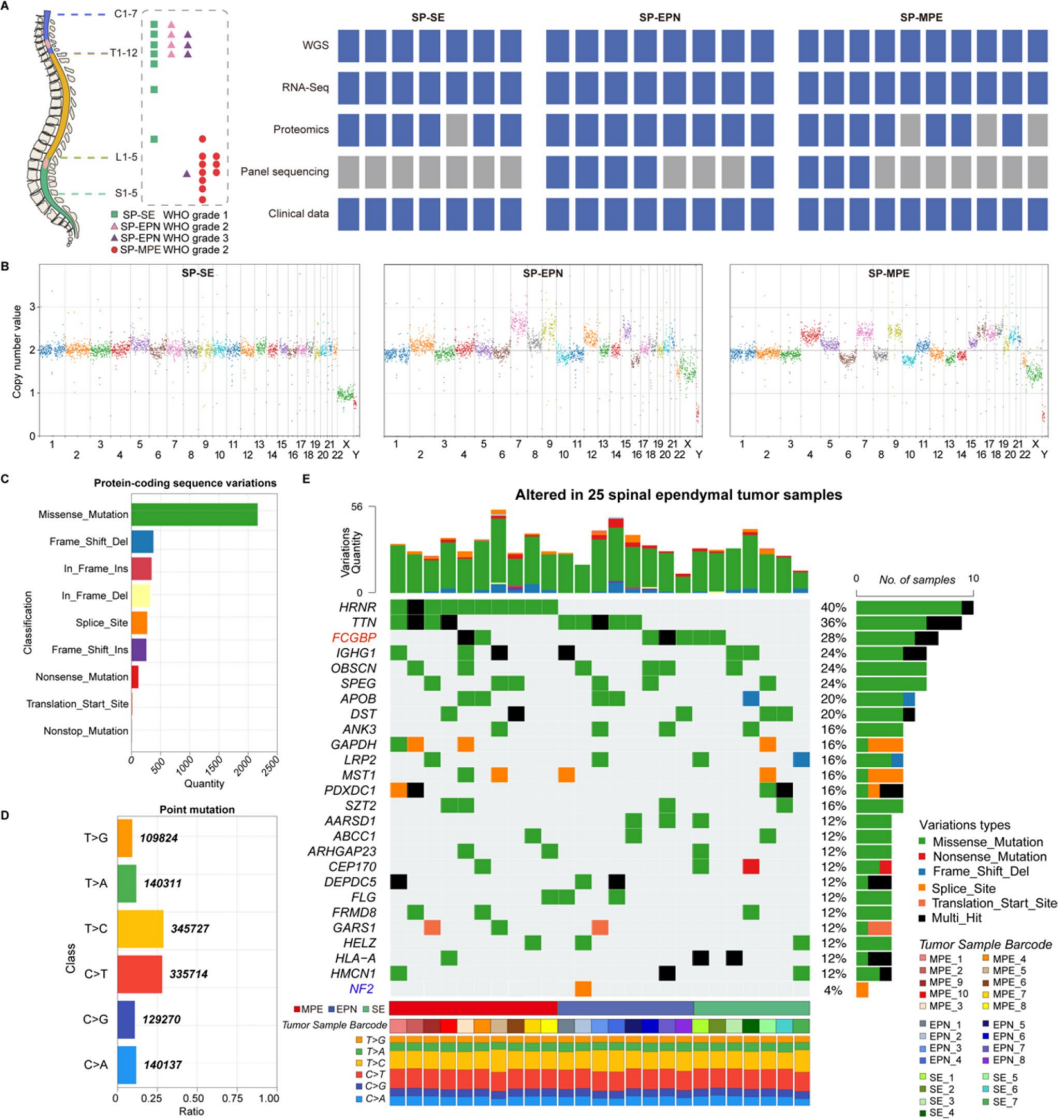
Genomic landscape of three subtypes of spinal ependymal tumors. (**A**), Summary of the spinal ependymal tumor cohort. Left panel, the anatomical distribution and pathological types of tumors in the spinal cord diagram. Right panel: The number of cases and subtypes in the cohorts, along with the types of data included (blue), with gray representing missing data. (**B**), Manhattan plot of the read depth signal in chromosome copy number alteration (CNA) profiles in the three types of spinal ependymal tumors. The bin size is 1Mb. (**C**), Nine types of protein-coding sequence variations were identified, with missense mutations being the most common. The number of variants was subdivided using the Human Genome Variation Society (HGVS) classification criteria [19]. (**D**), Types and quantities of point mutations, with the top two being T >C and C >T. (**E**), Potential mutated genes are ordered by *q* value. Each column denotes an individual sample, and each row represents a gene. Top panel, the protein-coding sequence variation events. Right panel: Percentage of mutations across 25 samples. Mutation types are shown by color as indicated. Genes annotated as “Multi Hit” have more than one type of mutation in the sample

To characterize the preliminary molecular features of spinal ependymal tumors, we conducted a comprehensive analysis that included WGS (*n* = 25), RNA sequencing (RNA-seq) (*n* = 25), targeted sequencing of brain tumor molecular markers (panel-seq) (*n* = 8), and proteomics analysis (*n* = 21) across the three distinct subtypes of spinal ependymal tumors (Fig. 1A). The clinicopathological and immunohistochemical characteristics of patients and their tumors are summarized in Tables S1-S3. Specifically, the cohort comprised 60% male and 40% female patients with a median age of 37 (Table S1). The histopathological classification of the cohort included 4 grade-2 and 4 grade-3 SP-EPN, seven grade-1 SP-SE, and ten grade-2 SP-MPE cases. All samples, except SP-EPN_7, were primary spinal ependymal tumors (Table S2). As shown in Table S3, Ki-67 levels vary significantly. Grade-3 SP-EPN exhibited a higher Ki-67 proliferation index compared to grade-2 ones. SP-SE and SP-MPE cases show a broader range, with some SP-MPE cases exhibiting higher Ki-67 levels (up to 15%). Variability in the expression of EMA and Olig-2 suggests the need for integrative analysis to refine molecular and clinical classification. To confirm the *MYCN*-status, we performed IHC staining using *MYCN* antibody (Cell Signaling Technolgy, N-Myc, D4B2Y, 1:100) and analysed *MYCN* copy number using WGS data for all the samples. Immunohistochemistry analysis revealed negative *MYCN* staining in all 8 SP-EPN samples (Supplementary Fig. 1). WGS analysis confirms that in cases SP-EPN3/4, WGS and panel sequencing revealed *MYCN* copy-number amplification, yet IHC staining of *MYCN* (Supplementary Fig. 1) was negative. This cohort contained three types ependymal tumors of the spinal cord, and all were from the Han Chinese population, which is distinct from previous spinal ependymal tumor multi-omics studies [6, 7].

### The CNAs patterns in spinal ependymal tumors

To obtain a comprehensive view of copy number alterations (CNAs) across all chromosomes, we performed WGS and used CNVpytor [14] to analyze CNAs in different spinal ependymal tumor subtypes (Supplementary table CNV).

In SP-SE, CNAs display a relatively balanced and random distribution, with no discernible focal amplifications or deletion hotspot regions (Fig. 1B left). Although a 6q deletion has been reported in brain SE [15], our analysis of seven SP-SE samples did not identify a similar trend, underscoring the variability and inconsistency of CNAs between the brain and spinal SE. By contrast, SP-MPE and SP-EPN displayed significantly higher CNAs. The most frequent event in SP-EPN tumors was the loss of 22 (Fig. 1B middle). Additionally, we observed copy number gains on chromosomes 7, 9, 12, and 15q in SP-EPN and on chromosomes 4, 7, 9, 16, 17, and 18 in SP-MPE (Fig. 1B right). The CNA burden in SP-SE was significantly lower than in SP-EPN and SP-MPE (Kruskal–Wallis test, *p* < 0.001; pairwise Wilcoxon test, adjusted *p* < 0.01). These findings demonstrate that spinal ependymal tumors across these three molecular subgroups are genetically distinct.

### The landscape of potential somatic mutations in spinal ependymal tumors

Next, we identified potential somatic mutations using MutSigCV algorithm [16] and compared them against the Catalogue of Somatic Mutations in Cancer (COSMIC), dbSNP databases and NyuWa Genome resource to filter SNPs and genetic variations prevalent in healthy people [17, 18]. Nine types of protein-coding sequence variations were detected. The top four protein-coding sequence variations were missense mutations, followed by frameshift deletions, in-frame insertions, and in-frame deletions (Fig. 1C). The most common point mutations observed were T >C, C >T, and T >A, with frequencies of 28.8%, 28.0%, and 11.7%, respectively (Fig. 1D). These point mutations did not show a preferential pattern toward specific subtypes of ependymal tumors (Fig. 1E, lower panel). The protein-coding sequence variation events across 25 samples ranged from 13 to 56 (Fig. 1E, upper panel). The top ten high-frequency protein-coding sequence variations occurred in HRNR, TTN, FCGBP, IGHG1, OBSCN, SPEG, APOB, DST, ANK3 and GAPDH (Fig. 1E, middle panel). Previous studies have shown that among the 21 SP-EPN analyzed, 19 exhibited a loss of 22q, where the *NF2* gene is located [6]. However, in our cohort, only one out of eight SP-EPNs carried an *NF2* mutation (Fig. 1E, highlighted in blue), indicating that the mutation frequency of this gene is correlated with the genetic background. Prior studies reported that 19/21 SP-EPNs exhibited 22q deletions spanning the *NF2* locus [6]. In our cohort, chromosome 22q deletion was observed in 4/8 (50%) SP-EPN cases. Notably, all four SP-EPN (SP-EPN_1–4) cases with 22q deletion are classified as histological grade 2 ependymomas (Wilcoxon test, *p* = 0.029). Meanwhile, *NF2* mutations were rare in our SP-EPN cohort (SP-EPN_2, Fig. 1E), the predominant occurrence of chromosome 22q deletion (4/8 cases) suggests that broader genomic loss across this chromosomal region, rather than isolated *NF2* inactivation, may underlie tumorigenesis in these subtypes.

### Different spinal ependymal tumor subtypes exhibit distinct features

**Fig. 2:**
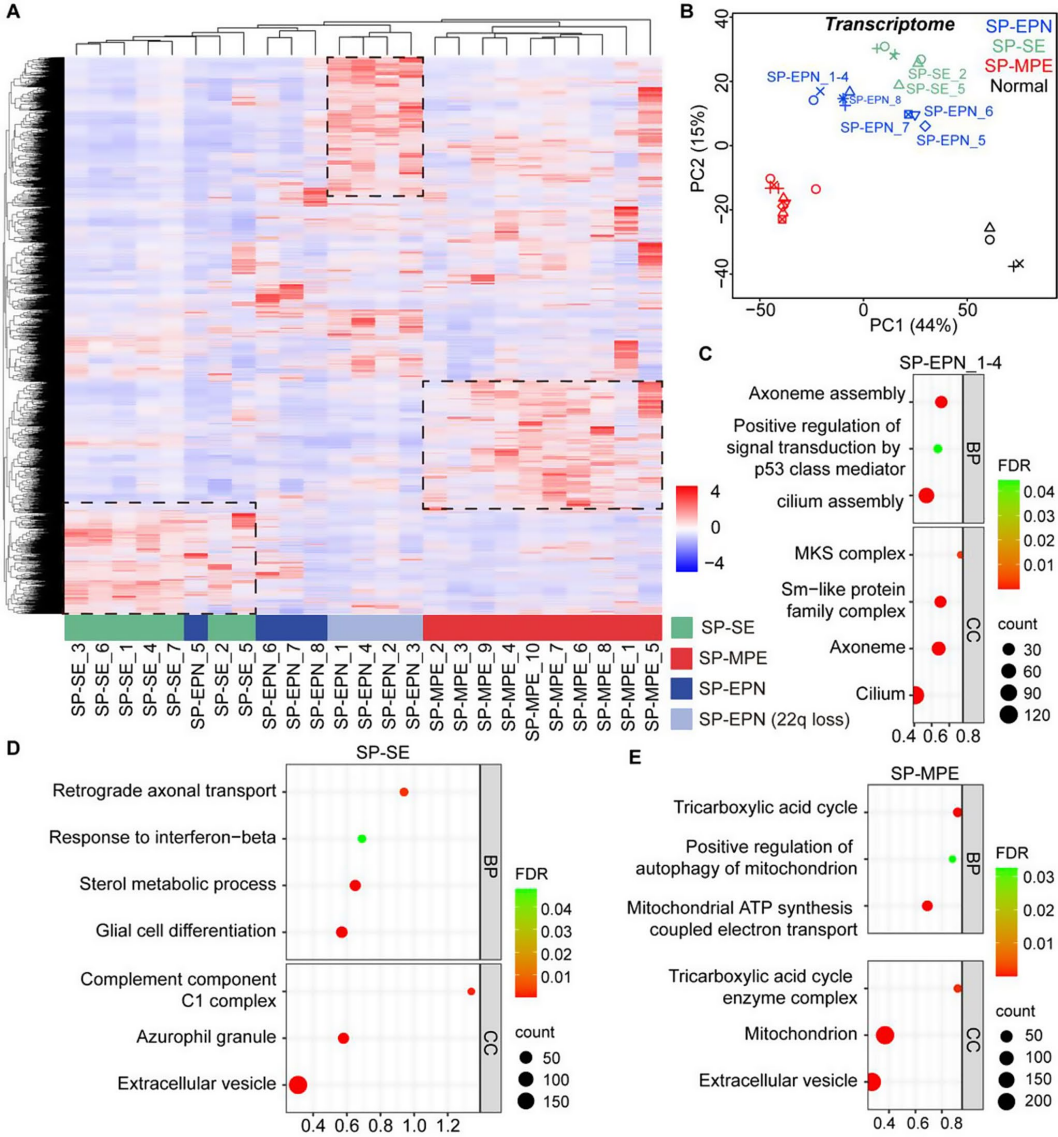
Transcriptomic Profiling Reveals Subtype-Specific Signatures in Spinal Ependymal Tumors. (**A**), Heatmap of transcriptomics among the three subtypes of spinal ependymal tumors (*n* = 25). (**B**), PCA analysis of transcriptomic data. **(C-E)**, GO analysis indicated the upregulated transcript enriched in pathways in SP-EPN (**C**), SP-SE (**D**), and SP-MPE (**E**)

To construct a transcriptomic map of various spinal ependymal tumor subtypes, we performed RNA-seq and conducted pairwise comparisons between the tumor samples. Our analysis revealed distinct transcriptional profiles for the SP-SE and SP-MPE subtypes across different patients, suggesting a unique molecular signature for each tumor type (Fig. 2A). Meanwhile, SP-EPN transcriptional patterns tended to cluster according to tumor grade. Specifically, grade-2 SP-EPN (SP-EPN_1/2/3/4) clustered together. The other three grade-3 SP-EPN (SP-EPN_6/7/8) showed a unique transcription pattern that differed from grade-2 EPN, suggesting that grade-3 SP-EPN is highly heterogeneous. These findings were confirmed by Principal Component Analysis (PCA) results, which aligned with those observed in the RNA-seq heatmap (Fig. 2B).

To gain deeper insights, we performed Gene Ontology (GO) and Kyoto Encyclopedia of Genes and Genomes (KEGG) pathway analyses of differentially expressed genes (DEGs), as highlighted by the black square in Fig. 2A. GO analysis revealed that the upregulated DEGs in SP-EPN_1/2/3/4 were primarily enriched in terms related to primary cilia, including axoneme assembly, cilium, and *MKS* complex (Fig. 2C). Primary cilia play crucial roles in signaling pathways during spinal development, such as Hh signaling [20, 21]. This finding is akin to Hedgehog pathway activation observed in supratentorial ependymomas, where primary cilia-dependent signaling confers oncogenic vulnerability [22]. Consistent with this, Gene Set Enrichment Analysis (GSEA) revealed significant enrichment of the Hh signaling pathway in grade-2 SP-EPN_1/2/3/4 (Supplementary Fig. 2A). The ciliary gene expression pattern may represent a remnant of the tumor’s ependymal lineage, independent of Hh pathway activity. While our computational analyses suggest a potential association between Hh signaling and SP-EPN oncogenesis, this link remains speculative without direct experimental validation.

In contrast, SP-SE tumors showed significant enrichment of immune-related response pathways, such as the response to interferon-related pathways and complement component C1 complex, as indicated by both GO and GSEA analyses (Fig. 2D, Supplementary Fig. 2B).

Transcriptomic profiling revealed enrichment of innate immune pathway signatures in SP-SE tumors. While these signatures may suggest tumor microenvironmental remodeling, bulk RNA-seq cannot distinguish between tumor-intrinsic pathway activation versus infiltrating immune cell contributions. Additionally, the enrichment of the complement component C1 complex points to the activation of the complement system, a key component of innate immunity that helps clear pathogens and modulate inflammation. This suggests that immune-related mechanisms may either support tumor progression in SP-SE or may reflect an attempt by the immune system to control tumor growth.

Interestingly, SP-MPE tumors exhibited preferential enrichment in mitochondrial and cellular metabolic pathways (Fig. 2E, Supplementary Fig. 2C). These include processes such as positive regulation of mitochondrial autophagy, mitochondrial ATP synthesis coupled to electron transport, and the tricarboxylic acid (TCA) cycle. These findings suggest that SP-MPE tumors have distinct metabolic characteristics that may be central to their pathophysiology. Notably, this metabolic profile aligns with features of the Warburg effect previously described in other malignancies and could support the increased energy demands of SP-MPE tumors, potentially contributing to their progression.

To identify potential biomarkers for the three distinct types of spinal ependymal tumors, we selected the genes that exhibited the most significant fold changes in transcript level. Our analysis revealed that *HOXB13*, *PRAC1*, and *SPARCL1* were preferentially expressed in SP-MPE, whereas APOE and LARP3 were highly expressed in SP-SE. *BANF*, *C9orf3*, *CAPS2*, *CALR3*, *C4orf50*, *CAPNS2* and *BCAR1* showed a preferential expression in grade-2 SP-EPN (Supplementary Fig. 3).

To identify potential onco-fusions in spinal ependymal tumors, we used Arriba to detect fusion transcripts. As a result, we identified *CTBS-GNG5* fusion genes in all SP-MPE samples. However, this fusion gene was also found in normal tissues of various types [23], suggesting that it may not be involved in the development of SP-MPE. Additionally, we identified *KIAA0319L-PARK7* fusion in SP-EPN_6 and *MAP7-MDFI* fusion in SP-SE_1 (Supplementary Fig. 4), although the functions of these two fusion genes remain unclear.

To rigorously validate the gene fusions identified in our study, we performed PCR Validation with Sanger Sequencing. For fusion events, we designed junction-spanning primers and performed RT-PCR and Sanger sequencing of the PCR products. The chromatograms confirmed the exact breakpoint sequences (chr6:136388393-chr6:41653319 for *MAP7-MDFI*, chr1:35554350-chr1:7984894 for *KIAA0319L-PARK7*).

We speculated that the fusion of *KIAA0319L* and *PARK7* results in a protein that integrates the endocytosis capabilities of *KIAA0319L* [24] with *PARK7*, an enzyme mutated in hereditary Parkinson’s disease, [25] potentially influencing SP-EPN_6 cellular responses to oxidative stress (Supplementary Fig. 4A). The breakpoint of MAP7-MDFI was located in the intron between exons 5 and 6 of *MAP7* and the intron between exons 4 and 5 of *MDFI* (Supplementary Fig. 4B). *MAP7* is a microtubule-binding protein [26] and *MDFI* is a transcription factor that negatively regulates other myogenic family proteins [27]. Thus, *MAP7-MDFI* fusion could potentially influence cellular architecture and differentiation processes in SP-SE_1.

### Proteomics profiling of spinal ependymal tumors

**Fig. 3:**
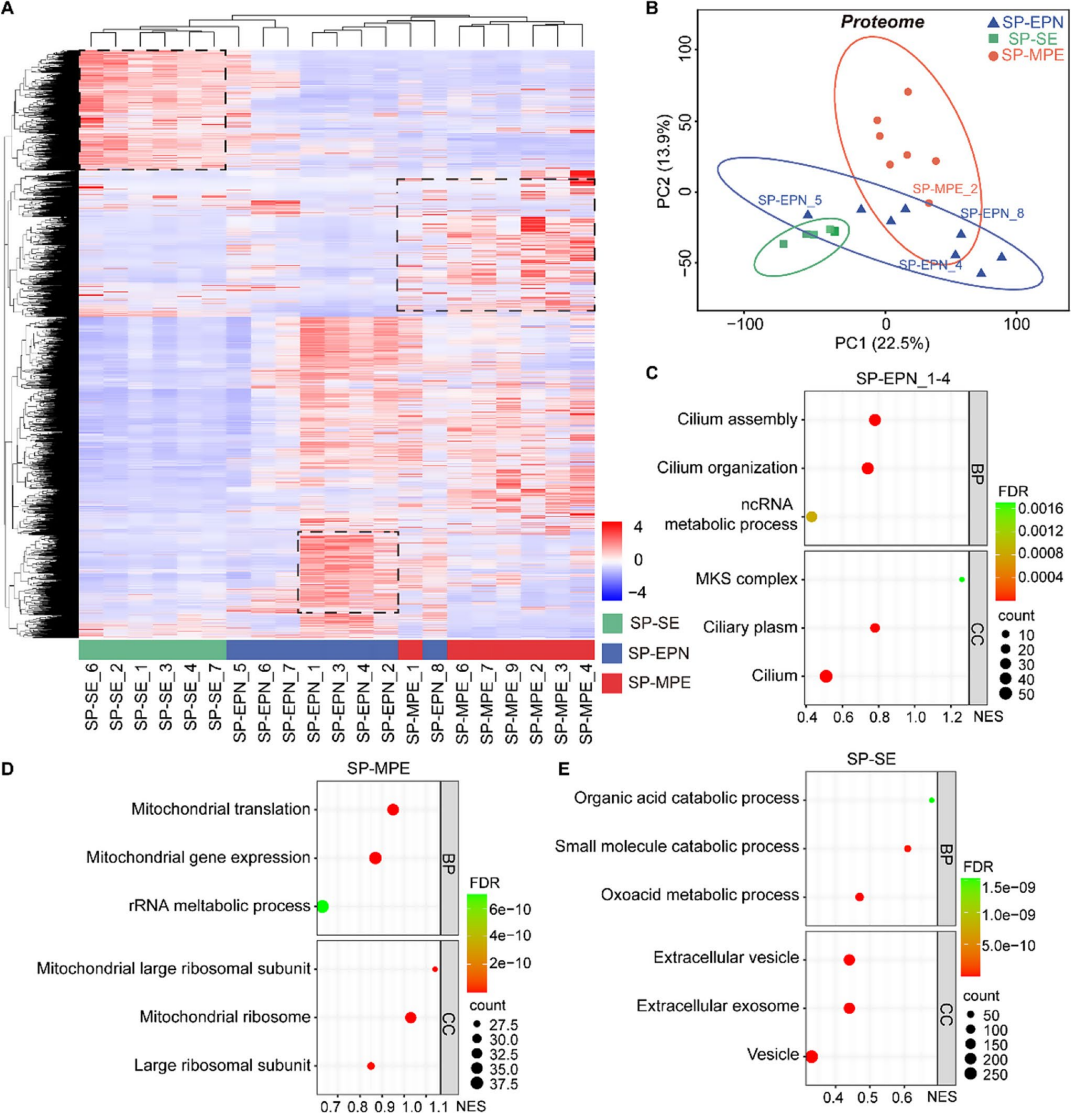
Proteomic characterization highlights molecular differences across spinal ependymal tumor subtypes. (**A**), Heatmap of proteomic among the three types of spinal ependymal tumors (*n* = 21). (**B**), PCA of proteomics data. (**C-E**), Pathways that were significantly upregulated in SP-EPN (**C**), SP-MPE (**D**), and SP-EPN (**E**)

To characterize the proteomic landscape of spinal ependymal tumors, we performed proteomic analyses on 21 samples, including 6 SP-SE, 8 SP-EPN, and 7 SP-MPE specimens. A total of 8,689 proteins were identified (Fig. 3A). PCA revealed that SP-EPN displayed characteristics intermediate between SP-SE and SP-MPE (Fig. 3B).

Differentially expressed proteins, highlighted within the black square in Fig. 3A, were subjected to GO analysis. In grade-2 SP-EPN subtypes (SP-EPN_1/2/3/4), upregulated proteins were enriched in cilia-related biological processes, including cilium assembly, cilium movement, and the *MKS* complex (Fig. 3C). In contrast, SP-MPE tumors were characterized by pronounced expression and translation of mitochondrial proteins, indicating a distinct metabolic profile unique to this subtype (Fig. 3D), aligning with the observations from RNA-seq analysis (Fig. 2).

For SP-SE tumors, GO analysis revealed significant enrichment in metabolic processes and extracellular vesicle-related pathways (Fig. 3E). This suggests that SP-SE tumors may harbor a unique tumor microenvironment modulated by extracellular vesicles. We hypothesize that these vesicles contribute to inflammatory factor secretion, as previously indicated by transcriptomic analyses of SP-SE tumors.

### Integrated analysis of spinal ependymal tumors

We combined the RNA-seq and proteomics results to characterize the three tumor subtypes. A total of 461 candidates exhibited consistent upregulation in both the transcriptomic and proteomic datasets, with 234, 134, and 93 candidates specifically upregulated in SP-SE, SP-EPN, and SP-MPE, respectively (Figure 5A-F). Grade-3 SP-EPN samples were excluded because of their heterogeneous transcriptomic and proteomic patterns.

**Fig. 4:**
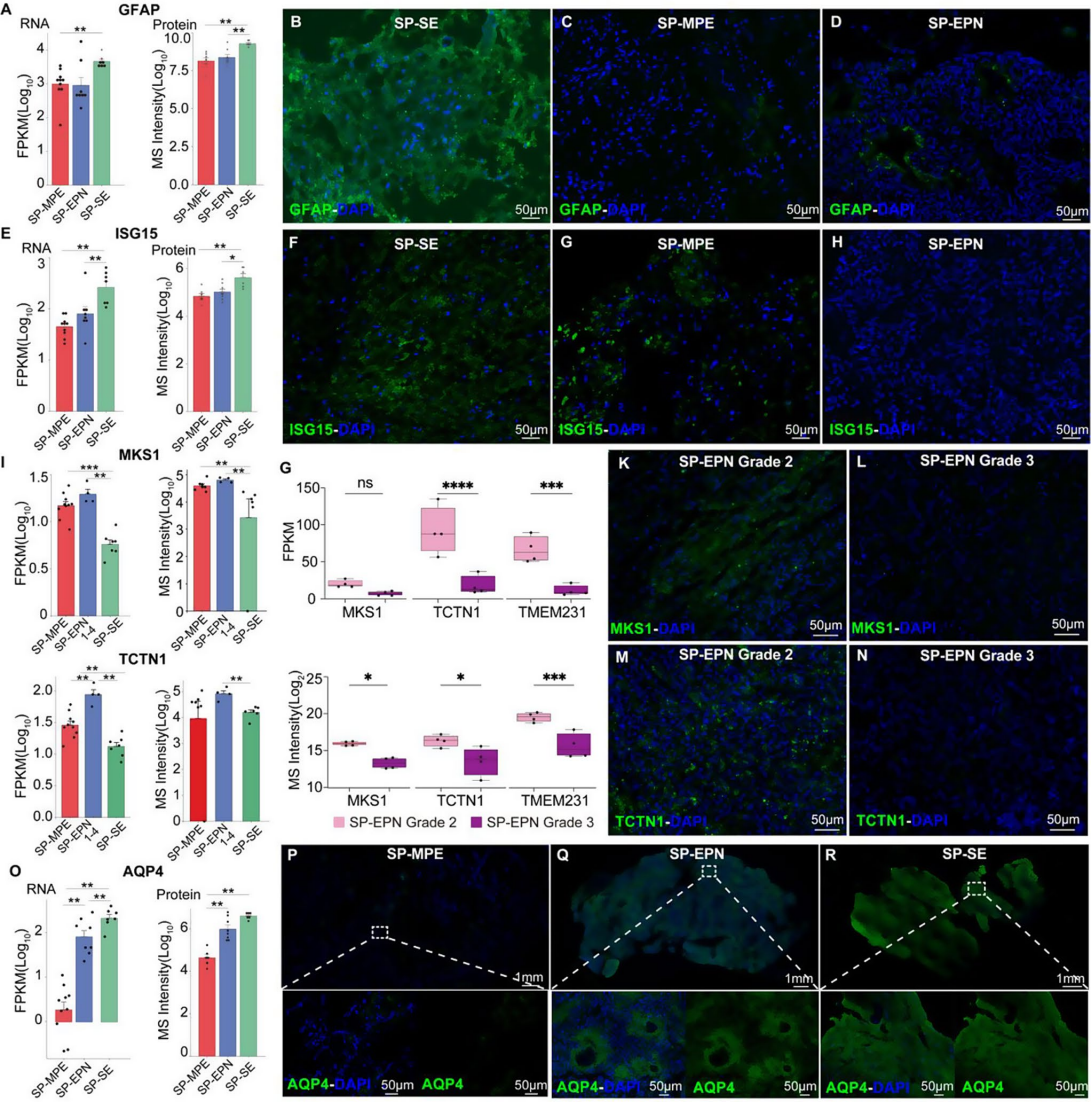
Integrative Proteogenomic Analysis and Immunofluorescence. **(A**,** E**,** I**,** O)**, The transcript (left) or protein abundance (right) of *GFAP* (A), *ISG15* (E), *MKS1*/*TCTN1* (I), and *AQP4* (O). **(B-D**,** F-H**,** P-R**), Representative IF staining images of *GFAP* (B-D), *ISG15* (F-H), and *AQP4* (P-R) in FFPE samples from three subtypes. Scale bar, 50 μm. **(G)**, Boxplots of differential expression of *MKS* complex genes based on RNA (top) and proteomic data (bottom). **(K-N)**, Representative IF staining images of *MKS1* (K, L) and *TCTN1* (M, N) in independent samples of grade-2 and grade-3 SP-EPN. *, *P* ≤ 0.05; **, *P* ≤ 0.01; ***, *P* ≤ 0.001; ns, *P* > 0.05

**Fig. 5:**
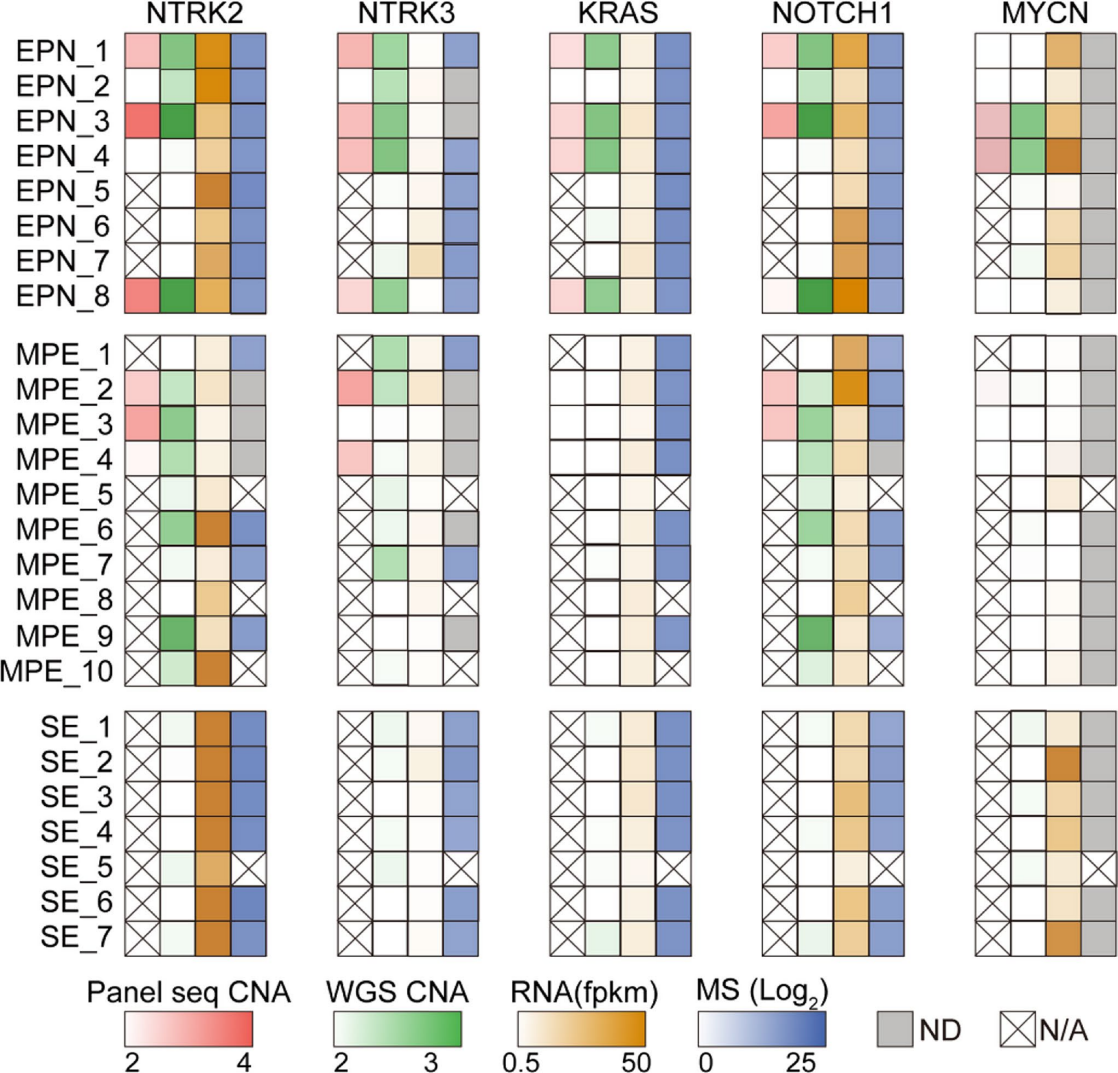
Integrated multi-omics analysis of five glioma-associated genes. The heatmap systematically integrates four molecular layers of five glioma-associated genes. DNA copy number variations (red and green panel) calculated via panel sequencing and WGS. Transcript abundance (brown panel) represented by RNA-seq FPKM values. Protein expression levels (blue panel) quantified through LC-MS/MS spectral intensity (log2-transformed). ND, Not detected; N/A, not available

In SP-SE, upregulated candidates were enriched in glial cell differentiation pathways, in addition to the previously identified extracellular vesicle pathways (Figure 5B). Among these, *GFAP* and *ISG15* drew particular attention (Fig. 4A and E). *GFAP*, a marker of glial differentiation [28, 29]. *ISG15*, an interferon-stimulated gene involved in immune pathways, plays an immunoregulatory role in tumors and serves as a potential biomarker for cancer progression [30, 31]. Although *GFAP* was ubiquitously expressed in 84% (21/25) of ependymoma samples across all subtypes (Table S3), IF staining of FFPE slices confirmed preferential expression of *GFAP* (Fig. 4B vs. C/D) and *ISG15* (Fig. 4F vs. G/H) in SP-SE.

In grade-2 SP-EPN, cilium-related genes were prominently expressed (Figure 5D). Among these, *MKS1* and *TCTN1*, key components of the *MKS* complex, showed strong positive correlations between RNA and protein levels (Pearson’s *R* = 0.68488 and *R* = 0.73098, respectively). Both genes demonstrated significantly higher expression in grade-2 SP-EPN compared to other subtypes (Fig. 4I). This finding was further validated using an independent SP-EPN cohort, where *MKS* complex-related genes, including *MKS1*, *TCTN1*, and *TMEM231*, were notably upregulated in grade-2 SP-EPN compared to grade-3 ones (Fig. 4G). Immunofluorescence staining confirmed these observations, showing high expression of *MKS1* (Fig. 4K vs. L) and *TCTN1* (Fig. 4M vs. N) in grade-2 SP-EPN, while their expression was markedly reduced in grade 3 SP-EPN.

The *MKS* complex, located in the primary ciliary transition zone, mediates essential developmental processes including ciliary formation and epithelial morphogenesis [32, 33].

Low expression of *TCTN1* expression the ciliary transition zone, compromises barrier function and impairs Hh signaling [34, 35]. This finding partially explained the enrichment of Hh signaling observed in our RNA-seq results.

In SP-MPE, upregulated candidates were enriched in mitochondrial translation pathways (sFigure 5 F). Using the STRING database, we constructed a PPI network of upregulated DEGs, highlighting mitochondrial ribosomal proteins (*MRPL58*, *MRPL15*, *MRPL17*, *MRPL37*, *MRPL16*, and *MRPS23*) as central nodes (yellow dots). We further analyzed genes downregulated in SP-MPE and focused on Aquaporin-4 (AQP4). Its downregulation in SP-MPE was validated by IF staining (Fig. 4P vs. Q/R). Previous studies suggested that *AQP4* participated in CNS water balances [36]. SP-MPE has been reported to present with cerebrospinal fluid dissemination or “drop metastases” at the time of diagnosis [37]. We speculated that *AQP4* depletion in SP-MPE may lead to cerebrospinal fluid imbalance, potentially contributing to the spread of SP-MPE to other locations via cerebrospinal fluid imbalance. Endocytosis-related proteins (*HIP1R*, *BIN1*, *FNBP1*, *DNM3*, *MYO6*, *NES* and *STON2*) were downregulated in SP-MPE (Figure 4G). Notably, *HIP1R* was almost absent in the SP-MPE group.

Among the 25 patients, eight underwent targeted sequencing based on brain glioma indices. However, the copy number amplifications detected in genes such as *NTRK2*, *NTRK3*, *KRAS*, and *NOTCH1* did not correlate with their RNA and protein expression levels. This suggests that gene amplification may not reliably represent transcriptional and proteomic changes in spinal ependymal tumors (Fig. 5).

## Discussion

Spinal ependymal tumors are highly heterogeneous, and understanding their molecular characteristics provides insight into their clinical behavior and potential therapeutic strategies.

### SP-EPN: abnormal ciliary signaling and therapeutic implications

In grade-2 SP-EPN tumors, we observed significant enrichment of ciliary signaling pathways, particularly those involving the *MKS* complex and Hh signaling. Dysregulation of ciliary components, such as TCTN1 and MKS1 may contribute to tumorigenesis through abnormal Hh pathway activation [38–40]. These findings suggest the potential therapeutic utility of targeting the Hh signaling pathway. These pathways require functional validation (e.g., spatial transcriptomics) to distinguish tumor-intrinsic immune activation from background biological noise. Drugs such as Vismodegib and Sonidegib, which inhibit Hh signaling, could be explored as adjunctive treatments for this subtype [41, 42]. In contrast, grade-3 SP-EPN tumors exhibit notable intertumor heterogeneity, indicating that additional clinical samples and further stratification are necessary to refine their molecular profiling.

### SP-MPE: mitochondrial metabolism and the Warburg effect

SP-MPE tumors demonstrate strong enrichment of mitochondrial pathways, including the TCA cycle, which distinguishes them from the other two subtypes. This is consistent with previous reports on the Warburg effect in SP-MPE [43]. Thus, targeting SP-MPE metabolism through inhibitors of TCA metabolism, such as glucose analog 2-deoxy-D-glucose [44], could be a promising therapeutic strategy for SP-MPE.

### SP-SE: immune-related pathways and extracellular vesicles

SP-SE tumors were enriched in immune-related pathways, particularly interferon signaling and extracellular vesicle-related genes. High expression of interferon-stimulated genes and extracellular vesicles indicates a unique tumor microenvironment characterized by active immune modulation.

Recurrent *HRNR* and *FCGBP* mutations observed in spinal cord ependymomas have also been reported in other tumor types. Studies have shown that the HRNR gene facilitates hepatocellular carcinoma progression and is associated with a poor prognosis [45]. The *HRNR* mutations identified in our study only occurred in SP-MPE. In non-small cell lung cancer, *FCGBP* exhibits the highest mutation frequency among smoking-related genes, with its downregulation strongly correlating with enhanced tumor proliferation, migration, and immunosuppressive microenvironments in smokers [46]. *FCGBP* mutations occurred at a frequency of 28% across all three ependymoma subtypes and the potential functional significance demanding further mechanistic study. In addition, *KIAA0319L-PARK7* and *MAP7-MDFI* are two novel fusions not reported in previous literature.

Our integrated transcriptomic and proteomic analysis revealed enrichment of cilia-related signaling pathways in SP-EPN and distinct metabolic features in SP-MPE. This finding aligns with the work of Gojo et al., [47] who systematically characterized pediatric posterior fossa ependymomas. They identified PF-ependymal-like cells characterized by ciliogenesis markers, alongside PF-metabolic and PF-immune-reactive metaprograms corresponding to glycolytic metabolism and immune effector functions, respectively.

Furthermore, our study detected aberrant *AQP4* expression in adult spinal ependymomas. This finding extends analogous subpopulations previously described in childhood ependymoma by Gillen et al., [48] who delineated ciliated EPN cells expressing ependymal differentiation markers and transportive EPN cells enriched with aquaporin transporters (specifically *AQP4*). Finally, consistent with our previous single-cell analyses [49], upregulation of the Hedgehog pathway and epithelial-mesenchymal transition (EMT) signatures was observed in SP-EPN. While further validation is required, the convergence of these molecular features across independent studies suggests potential fundamental commonalities in ependymoma pathogenesis.

The small sample size and single-center design limit the population-level generalizability of these findings. Subgroup analyses should be interpreted as hypothesis-generating rather than definitive characterizations. While multi-omics integration remains a key strength, the absence of DNA methylation data in this study prevents direct comparison with prior studies.

## Conclusion

This study provides a comprehensive characterization of the molecular heterogeneity of spinal ependymal tumors. Grade-2 SP-EPN tumors with enriched ciliary signaling may benefit from targeted therapies such as Hh pathway inhibitors, while the metabolic reprogramming of SP-MPE suggests therapeutic opportunities for disrupting mitochondrial or Warburg pathway metabolism. Future studies with larger cohorts are essential to validate these findings and further refine subtype-specific therapeutic strategies.

## Supplementary Information

Below is the link to the electronic supplementary material.

Supplementary Material 1

Supplementary Material 2

Supplementary Material 3

Supplementary Material 4

Supplementary Material 5

Supplementary Material 6

Supplementary Material 7

Supplementary Material 8

Supplementary Material 9

Supplementary Material 10

Supplementary Material 11

## Data Availability

Raw WGS and RNA-seq data were deposited in the China National Center for Bioinformation (https://ngdc.cncb.ac.cn/gsa-human/) under the accession number HRA008375. The mass spectrometry proteomics data have been deposited to the ProteomeXchange Consortium (https://proteomecentral.proteomexchange.org) via the iProX partner repository with the dataset identifier PXD056112.

## References

[1] Q.T. Ostrom, G. Cioffi, H. Gittleman, N. Patil, K. Waite, C. Kruchko, J.S. Barnholtz-Sloan, CBTRUS statistical report: primary brain and other central nervous system tumors diagnosed in the united States in 2012–2016. Neuro Oncol. **21**, v1–v100 (2019). 10.1093/neuonc/noz150 31675094 10.1093/neuonc/noz150 PMC6823730

[2] F. Hanbali, D.R. Fourney, E. Marmor, D. Suki, L.D. Rhines, J.S. Weinberg, I.E. McCutcheon, I. Suk, Z.L. Gokaslan, Spinal cord ependymoma: radical surgical resection and outcome. Neurosurgery. **51**, 1162–1172 (2002). discussion 1172 – 1164. 10.1097/00006123-200211000-00010 12383361 10.1097/00006123-200211000-00010

[3] D.R. Gomez, B.T. Missett, W.M. Wara, K.R. Lamborn, M.D. Prados, S. Chang, M.S. Berger, D.A. Haas-Kogan, High failure rate in spinal ependymomas with long-term follow-up. Neuro Oncol. **7**, 254–259 (2005). 10.1215/S1152851704001231 16053700 10.1215/S1152851704001231 PMC1871913

[4] A.H. Saleh, N. Samuel, K. Juraschka, M.H. Saleh, M.D. Taylor, M.G. Fehlings, The biology of ependymomas and emerging novel therapies. Nat. Rev. Cancer. **22**, 208–222 (2022). 10.1038/s41568-021-00433-2 35031778 10.1038/s41568-021-00433-2

[5] S. Gritsch, T.T. Batchelor, L.N. Gonzalez Castro, Diagnostic, therapeutic, and prognostic implications of the 2021 world health organization classification of tumors of the central nervous system. Cancer. **128**, 47–58 (2022). 10.1002/cncr.33918 34633681 10.1002/cncr.33918

[6] K.W. Pajtler, H. Witt, M. Sill, D.T. Jones, V. Hovestadt, F. Kratochwil, K. Wani, R. Tatevossian, C. Punchihewa, P. Johann et al., Molecular classification of ependymal tumors across all CNS Compartments, histopathological Grades, and age groups. Cancer Cell. **27**, 728–743 (2015). 10.1016/j.ccell.2015.04.002 25965575 10.1016/j.ccell.2015.04.002 PMC4712639

[7] S. Neyazi, E. Yamazawa, K. Hack, S. Tanaka, G. Nagae, C. Kresbach, T. Umeda, A. Eckhardt, K. Tatsuno, L. Pohl et al., Transcriptomic and epigenetic dissection of spinal ependymoma (SP-EPN) identifies clinically relevant subtypes enriched for tumors with and without NF2 mutation. Acta Neuropathol. **147**, 22 (2024). 10.1007/s00401-023-02668-9 38265489 10.1007/s00401-023-02668-9 PMC10808175

[8] D.R. Ghasemi, M. Sill, K. Okonechnikov, A. Korshunov, S. Yip, P.W. Schutz, D. Scheie, A. Kruse, P.N. Harter, M. Kastelan et al., MYCN amplification drives an aggressive form of spinal ependymoma. Acta Neuropathol. **138**, 1075–1089 (2019). 10.1007/s00401-019-02056-2 31414211 10.1007/s00401-019-02056-2 PMC6851394

[9] M. Raffeld, Z. Abdullaev, S.D. Pack, L. Xi, S. Nagaraj, N. Briceno, E. Vera, S. Pittaluga, L. Abath Neto, O. Quezado, M., et al., High level MYCN amplification and distinct methylation signature define an aggressive subtype of spinal cord ependymoma. Acta Neuropathol. Commun. **8**, 101 (2020). 10.1186/s40478-020-00973-y 32641156 10.1186/s40478-020-00973-y PMC7346356

[10] M. Bockmayr, K. Harnisch, L.C. Pohl, L. Schweizer, T. Mohme, M. Korner, M. Alawi, A.K. Suwala, M.M. Dorostkar, C.M. Monoranu et al., Comprehensive profiling of myxopapillary ependymomas identifies a distinct molecular subtype with relapsing disease. Neuro Oncol. **24**, 1689–1699 (2022). 10.1093/neuonc/noac088 35380708 10.1093/neuonc/noac088 PMC9527524

[11] S. Uhrig, J. Ellermann, T. Walther, P. Burkhardt, M. Frohlich, B. Hutter, U.H. Toprak, O. Neumann, A. Stenzinger, C. Scholl et al., Accurate and efficient detection of gene fusions from RNA sequencing data. Genome Res. **31**, 448–460 (2021). 10.1101/gr.257246.119 33441414 10.1101/gr.257246.119 PMC7919457

[12] T. Aramaki, R. Blanc-Mathieu, H. Endo, K. Ohkubo, M. Kanehisa, S. Goto, H. Ogata, KofamKOALA: KEGG ortholog assignment based on profile HMM and adaptive score threshold. Bioinformatics. **36**, 2251–2252 (2020). 10.1093/bioinformatics/btz859 31742321 10.1093/bioinformatics/btz859 PMC7141845

[13] G. Yu, L.G. Wang, Y. Han, Q.Y. He, ClusterProfiler: an R package for comparing biological themes among gene clusters. OMICS. **16**, 284–287 (2012). 10.1089/omi.2011.0118 22455463 10.1089/omi.2011.0118 PMC3339379

[14] M. Suvakov, A. Panda, C. Diesh, I. Holmes, A. Abyzov, CNVpytor: a tool for copy number variation detection and analysis from read depth and allele imbalance in whole-genome sequencing. Gigascience. **10** (2021). 10.1093/gigascience/giab074 10.1093/gigascience/giab074 PMC8612020 34817058

[15] C. Thomas, F. Thierfelder, M. Trager, P. Soschinski, M. Muther, D. Edelmann, A. Forster, C. Geiler, H.Y. Kim, K. Filipski et al., TERT promoter mutation and chromosome 6 loss define a high-risk subtype of ependymoma evolving from posterior fossa subependymoma. Acta Neuropathol. **141**, 959–970 (2021). 10.1007/s00401-021-02300-8 33755803 10.1007/s00401-021-02300-8 PMC8113189

[16] M. Reich, T. Liefeld, J. Gould, J. Lerner, P. Tamayo, J.P. Mesirov, GenePattern 2.0. Nat. Genet. **38**, 500–501 (2006). 10.1038/ng0506-500 16642009 10.1038/ng0506-500

[17] J.G. Tate, S. Bamford, H.C. Jubb, Z. Sondka, D.M. Beare, N. Bindal, H. Boutselakis, C.G. Cole, C. Creatore, E. Dawson et al., COSMIC: the catalogue of somatic mutations in cancer. Nucleic Acids Res. **47**, D941–D947 (2019). 10.1093/nar/gky1015 30371878 10.1093/nar/gky1015 PMC6323903

[18] P. Zhang, H. Luo, Y. Li, Y. Wang, J. Wang, Y. Zheng, Y. Niu, Y. Shi, H. Zhou, T. Song et al., NyuWa genome resource: A deep whole-genome sequencing-based variation profile and reference panel for the Chinese population. Cell. Rep. **37**, 110017 (2021). 10.1016/j.celrep.2021.110017 34788621 10.1016/j.celrep.2021.110017

[19] den J.T. Dunnen, R. Dalgleish, D.R. Maglott, R.K. Hart, M.S. Greenblatt, J. McGowan-Jordan, A.F. Roux, T. Smith, S.E. Antonarakis, P.E. Taschner, HGVS recommendations for the description of sequence variants: 2016 update. Hum. Mutat. **37**, 564–569 (2016). 10.1002/humu.22981 26931183 10.1002/humu.22981

[20] R. Ma, N.A. Kutchy, L. Chen, D.D. Meigs, G. Hu, Primary cilia and ciliary signaling pathways in aging and age-related brain disorders. Neurobiol. Dis. **163**, 105607 (2022). 10.1016/j.nbd.2021.105607 34979259 10.1016/j.nbd.2021.105607 PMC9280856

[21] E.K. Ho, T. Stearns, Hedgehog signaling and the primary cilium: implications for spatial and temporal constraints on signaling. Development. **148** (2021). 10.1242/dev.195552 10.1242/dev.195552 PMC8126410 33914866

[22] de T. Almeida Magalhaes, A. Veiga Cruzeiro, G. Ribeiro de Sousa, G. Englinger, B.F.P. Nagano, L. Ancliffe, M.R. da Silva, K. Jiang, L. Gojo, J.C. Liu, Y., et al., Activation of Hedgehog signaling by the oncogenic RELA fusion reveals a primary cilia-dependent vulnerability in supratentorial ependymoma. Neuro Oncol. **25**, 185–198 (2023). 10.1093/neuonc/noac147 35640920 10.1093/neuonc/noac147 PMC9825332

[23] M. Babiceanu, F. Qin, Z. Xie, Y. Jia, K. Lopez, N. Janus, L. Facemire, S. Kumar, Y. Pang, Y. Qi et al., Recurrent chimeric fusion RNAs in non-cancer tissues and cells. Nucleic Acids Res. **44**, 2859–2872 (2016). 10.1093/nar/gkw032 26837576 10.1093/nar/gkw032 PMC4824105

[24] S. Pillay, N.L. Meyer, A.S. Puschnik, O. Davulcu, J. Diep, Y. Ishikawa, L.T. Jae, J.E. Wosen, C.M. Nagamine, M.S. Chapman, J.E. Carette, An essential receptor for adeno-associated virus infection. Nature. **530**, 108–112 (2016). 10.1038/nature16465 26814968 10.1038/nature16465 PMC4962915

[25] I.P. Heremans, F. Caligiore, I. Gerin, M. Bury, M. Lutz, J. Graff, V. Stroobant, D. Vertommen, A.A. Teleman, Van E. Schaftingen, G.T. Bommer, Parkinson’s disease protein PARK7 prevents metabolite and protein damage caused by a glycolytic metabolite. Proc Natl Acad Sci U S A . **119** (2022). 10.1073/pnas.2111338119 10.1073/pnas.2111338119 PMC8795555 35046029

[26] D. Masson, T.E. Kreis, Identification and molecular characterization of E-MAP-115, a novel microtubule-associated protein predominantly expressed in epithelial cells. J. Cell. Biol. **123**, 357–371 (1993). 10.1083/jcb.123.2.357 8408219 10.1083/jcb.123.2.357 PMC2119845

[27] C.M. Chen, N. Kraut, M. Groudine, H. Weintraub, I-mf, a novel myogenic repressor, interacts with members of the myod family. Cell. **86**, 731–741 (1996). 10.1016/s0092-8674(00)80148-8 8797820 10.1016/s0092-8674(00)80148-8

[28] Z. Yang, K.K. Wang, Glial fibrillary acidic protein: from intermediate filament assembly and gliosis to neurobiomarker. Trends Neurosci. **38**, 364–374 (2015). 10.1016/j.tins.2015.04.003 25975510 10.1016/j.tins.2015.04.003 PMC4559283

[29] van E.J. Bodegraven, van J.V. Asperen, P.A.J. Robe, E.M. Hol, Importance of GFAP isoform-specific analyses in Astrocytoma. Glia. **67**, 1417–1433 (2019). 10.1002/glia.23594 30667110 10.1002/glia.23594 PMC6617972

[30] W.M. Schneider, M.D. Chevillotte, C.M. Rice, Interferon-stimulated genes: a complex web of host defenses. Annu. Rev. Immunol. **32**, 513–545 (2014). 10.1146/annurev-immunol-032713-120231 24555472 10.1146/annurev-immunol-032713-120231 PMC4313732

[31] H.M. Nguyen, S. Gaikwad, M. Oladejo, M.Y. Agrawal, S.K. Srivastava, L.M. Wood, Interferon stimulated gene 15 (ISG15) in cancer: an update. Cancer Lett. **556**, 216080 (2023). 10.1016/j.canlet.2023.216080 36736853 10.1016/j.canlet.2023.216080

[32] F.R. Garcia-Gonzalo, K.C. Corbit, M.S. Sirerol-Piquer, G. Ramaswami, E.A. Otto, T.R. Noriega, A.D. Seol, J.F. Robinson, C.L. Bennett, D.J. Josifova et al., A transition zone complex regulates mammalian ciliogenesis and ciliary membrane composition. Nat. Genet. **43**, 776–784 (2011). 10.1038/ng.891 21725307 10.1038/ng.891 PMC3145011

[33] H.R. Dawe, U.M. Smith, A.R. Cullinane, D. Gerrelli, P. Cox, J.L. Badano, S. Blair-Reid, N. Sriram, N. Katsanis, T. Attie-Bitach et al., The Meckel-Gruber syndrome proteins MKS1 and Meckelin interact and are required for primary cilium formation. Hum. Mol. Genet. **16**, 173–186 (2007). 10.1093/hmg/ddl459 17185389 10.1093/hmg/ddl459

[34] L. Wang, X. Wen, Z. Wang, Z. Lin, C. Li, H. Zhou, H. Yu, Y. Li, Y. Cheng, Y. Chen et al., Ciliary transition zone proteins coordinate ciliary protein composition and ectosome shedding. Nat. Commun. **13**, 3997 (2022). 10.1038/s41467-022-31751-0 35810181 10.1038/s41467-022-31751-0 PMC9271036

[35] H.M. Truong, K.O. Cruz-Colon, J.Y. Martinez-Marquez, J.R. Willer, A.M. Travis, S.K. Biswas, W.K. Lo, H.J. Bolz, J.N. Pearring, The tectonic complex regulates membrane protein composition in the photoreceptor cilium. Nat. Commun. **14**, 5671 (2023). 10.1038/s41467-023-41450-z 37704658 10.1038/s41467-023-41450-z PMC10500017

[36] M.K. Oklinski, M.T. Skowronski, A. Skowronska, M. Rutzler, K. Norgaard, J.D. Nieland, T.H. Kwon, S. Nielsen, Aquaporins in the spinal cord. Int. J. Mol. Sci. **17** (2016). 10.3390/ijms17122050 10.3390/ijms17122050 PMC5187850 27941618

[37] J.K. Tabor, B. Ryu, D. Schneider, D.M. Sciubba, A. Narayana, A. Zlochower, R.S. Amico, Multifocal lumbar myxopapillary ependymoma presenting with drop metastasis: a case report and review of the literature. Spinal Cord Ser. Cases. **8**, 43 (2022). 10.1038/s41394-022-00513-x 35459220 10.1038/s41394-022-00513-x PMC9033832

[38] T. Kubota, J. Ishise, T. Yamashima, S. Yamamoto, Abnormal cilia in a malignant ependymoma. Acta Neuropathol. **71**, 100–105 (1986). 10.1007/BF00687969 3776464 10.1007/BF00687969

[39] S.C. Goetz, F. Bangs, C.L. Barrington, N. Katsanis, K.V. Anderson, The Meckel syndrome- associated protein MKS1 functionally interacts with components of the BBSome and IFT complexes to mediate ciliary trafficking and Hedgehog signaling. PLoS One. **12**, e0173399 (2017). 10.1371/journal.pone.0173399 28291807 10.1371/journal.pone.0173399 PMC5349470

[40] G. Wheway, L. Nazlamova, J.T. Hancock, Signaling through the primary cilium. Front. Cell. Dev. Biol. **6**, 8 (2018). 10.3389/fcell.2018.00008 29473038 10.3389/fcell.2018.00008 PMC5809511

[41] R. Dummer, A. Guminksi, R. Gutzmer, J.T. Lear, K.D. Lewis, A.L.S. Chang, P. Combemale, L. Dirix, M. Kaatz, R. Kudchadkar et al., Long-term efficacy and safety of sonidegib in patients with advanced basal cell carcinoma: 42-month analysis of the phase II randomized, double-blind BOLT study. Br. J. Dermatol. **182**, 1369–1378 (2020). 10.1111/bjd.18552 31545507 10.1111/bjd.18552 PMC7318253

[42] P.M. LoRusso, C.M. Rudin, J.C. Reddy, R. Tibes, G.J. Weiss, M.J. Borad, C.L. Hann, J.R. Brahmer, I. Chang, W.C. Darbonne et al., Phase I trial of Hedgehog pathway inhibitor vismodegib (GDC-0449) in patients with refractory, locally advanced or metastatic solid tumors. Clin. Cancer Res. **17**, 2502–2511 (2011). 10.1158/1078-0432.CCR-10-2745 21300762 10.1158/1078-0432.CCR-10-2745 PMC5244484

[43] S.C. Mack, S. Agnihotri, K.C. Bertrand, X. Wang, D.J. Shih, H. Witt, N. Hill, K. Zayne, M. Barszczyk, V. Ramaswamy et al., Spinal myxopapillary ependymomas demonstrate a Warburg phenotype. Clin. Cancer Res. **21**, 3750–3758 (2015). 10.1158/1078-0432.CCR-14-2650 25957288 PMC4537825

[44] D. Zhang, J. Li, F. Wang, J. Hu, S. Wang, Y. Sun, 2-Deoxy-D-glucose targeting of glucose metabolism in cancer cells as a potential therapy. Cancer Lett. **355**, 176–183 (2014). 10.1016/j.canlet.2014.09.003 25218591 10.1016/j.canlet.2014.09.003

[45] S.J. Fu, S.L. Shen, S.Q. Li, Y.P. Hua, W.J. Hu, B. Guo, B.G. Peng, Hornerin promotes tumor progression and is associated with poor prognosis in hepatocellular carcinoma. BMC Cancer. **18** (2018). 10.1186/s12885-018-4719-5 10.1186/s12885-018-4719-5 PMC6090597 30103712

[46] Q. Li, T. Wang, Y. Tang, X. Zou, Z. Shen, Z. Tang, Y. Zhou, J. Shi, A novel prognostic signature based on smoking-associated genes for predicting prognosis and immune microenvironment in NSCLC smokers. Cancer Cell. Int. **24**, 171 (2024). 10.1186/s12935-024-03347-9 38750571 10.1186/s12935-024-03347-9 PMC11094918

[47] J. Gojo, B. Englinger, L. Jiang, J.M. Hubner, M.L. Shaw, O.A. Hack, S. Madlener, D. Kirchhofer, I. Liu, J. Pyrdol et al., Single-Cell RNA-Seq reveals cellular hierarchies and impaired developmental trajectories in pediatric ependymoma. Cancer Cell. **38**, 44–59e49 (2020). 10.1016/j.ccell.2020.06.004 32663469 10.1016/j.ccell.2020.06.004 PMC7479515

[48] A.E. Gillen, K.A. Riemondy, V. Amani, A.M. Griesinger, A. Gilani, S. Venkataraman, K. Madhavan, E. Prince, B. Sanford, T.C. Hankinson et al., Single-Cell RNA sequencing of childhood ependymoma reveals neoplastic cell subpopulations that impact molecular classification and etiology. Cell. Rep. **32**, 108023 (2020). 10.1016/j.celrep.2020.108023 32783945 10.1016/j.celrep.2020.108023 PMC7452755

[49] Q. Zhang, S. Cheng, Y. Wang, M. Wang, Y. Lu, Z. Wen, Y. Ge, Q. Ma, Y. Chen, Y. Zhang et al., Interrogation of the microenvironmental landscape in spinal ependymomas reveals dual functions of tumor-associated macrophages. Nat. Commun. **12**, 6867 (2021). 10.1038/s41467-021-27018-9 34824203 10.1038/s41467-021-27018-9 PMC8617028

