# Supplementary material for: Multi-omics analysis delineates molecular signatures of spinal ependymal tumor
Source: Cell Oncol (Dordr). 2025 Oct 29;48(6):1987–2000. doi: 10.1007/s13402-025-01122-0 (PMC12698791; doi:10.1007/s13402-025-01122-0)
Supplement: Supplementary file 1 — Supplementary Material 1 [file 13402_2025_1122_MOESM1_ESM.docx]

**Supplementary** **
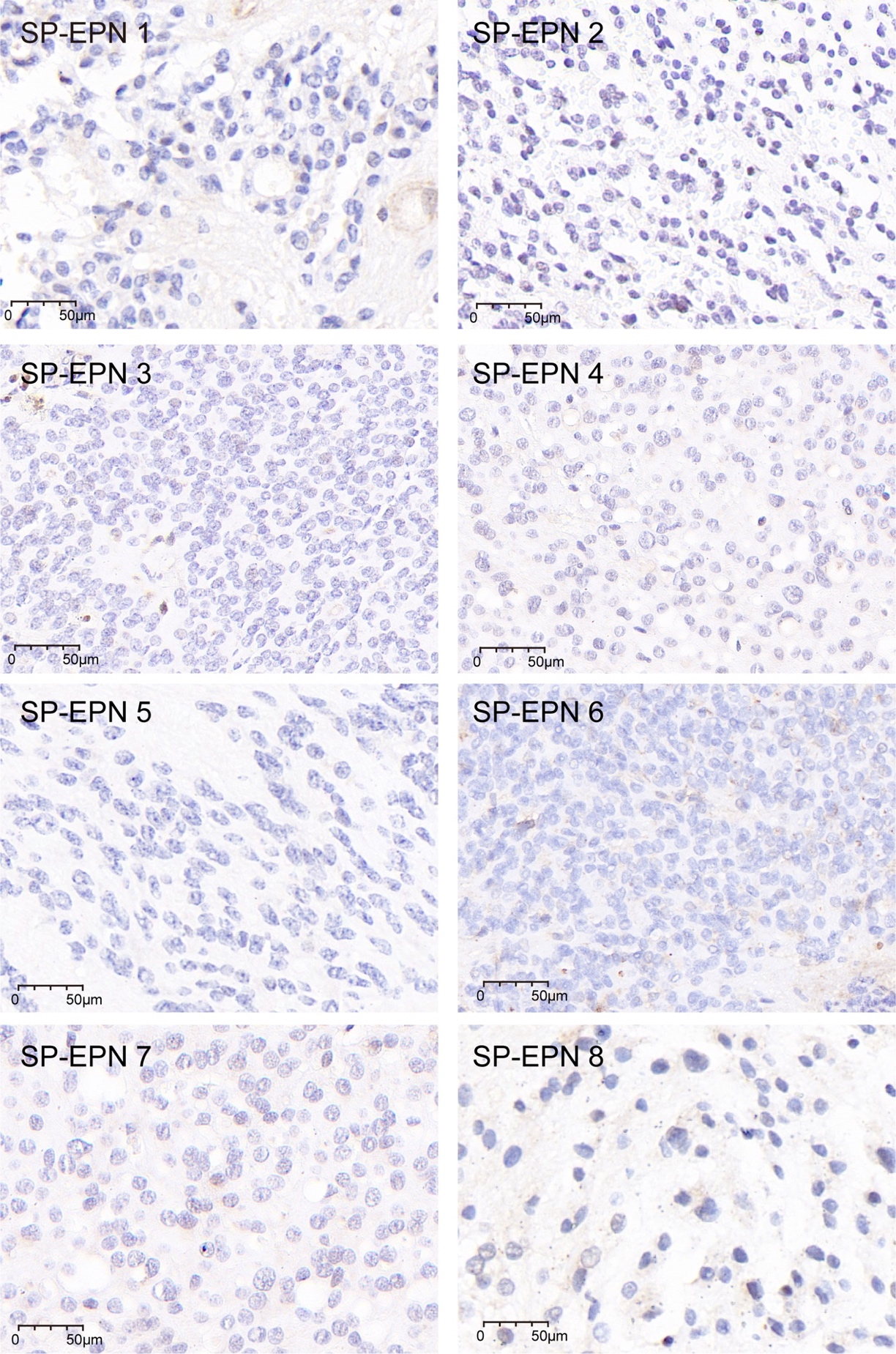
Figure 1. Immunohistochemical of *MYCN* in spinal cord ependymomas**

Immunohistochemical results of all SP-EPN samples demonstrated negative *MYCN* expression in the tumor tissue, with complete absence of nuclear staining in neoplastic cells (400x).

**
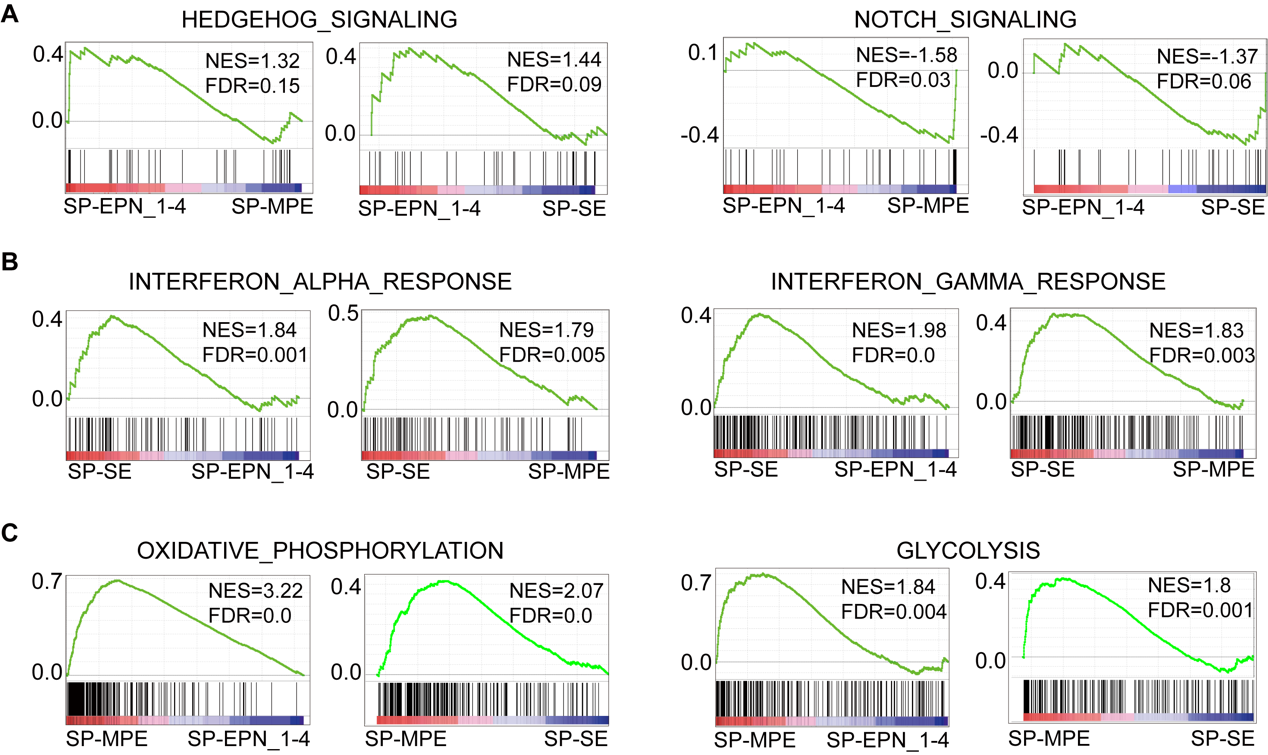
****Supplementary Figure 2. GSEA** **analysis of spinal ependymal tumors.**

GSEA plots for representative gene sets of SP-EPN 1-4 (A) SP-SE (B) and SP-MPE (C). The y-axis represents the enrichment score (ES) and the x-axis represents genes (vertical black lines) present in the gene sets. NES, normalized enrichment score; FDR, false discovery rate.

**
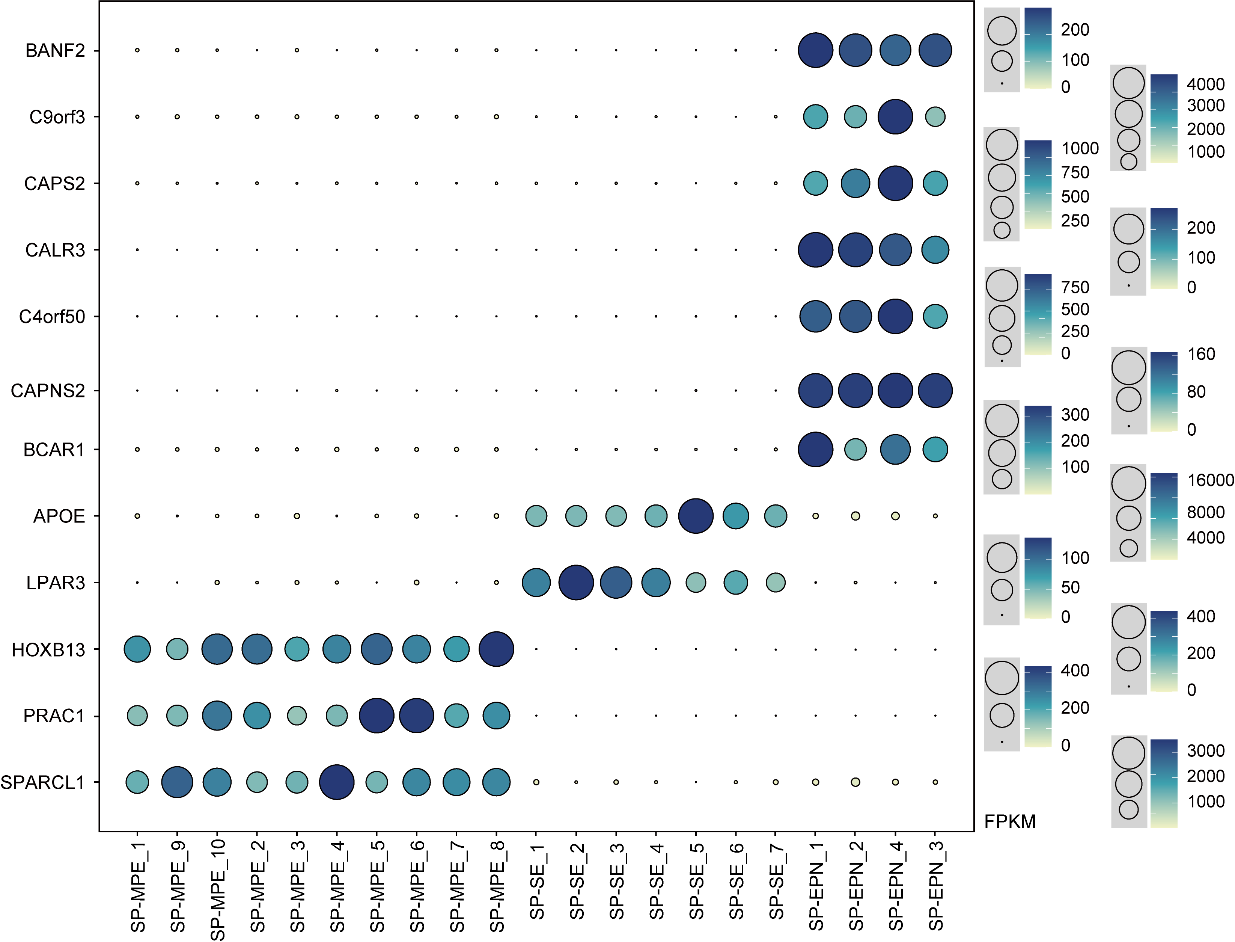
Supplementary Figure 3.** **Balloon plot of representative DEGs in transcriptome.**

The balloon plot quantifies the top different genes across three subtypes.


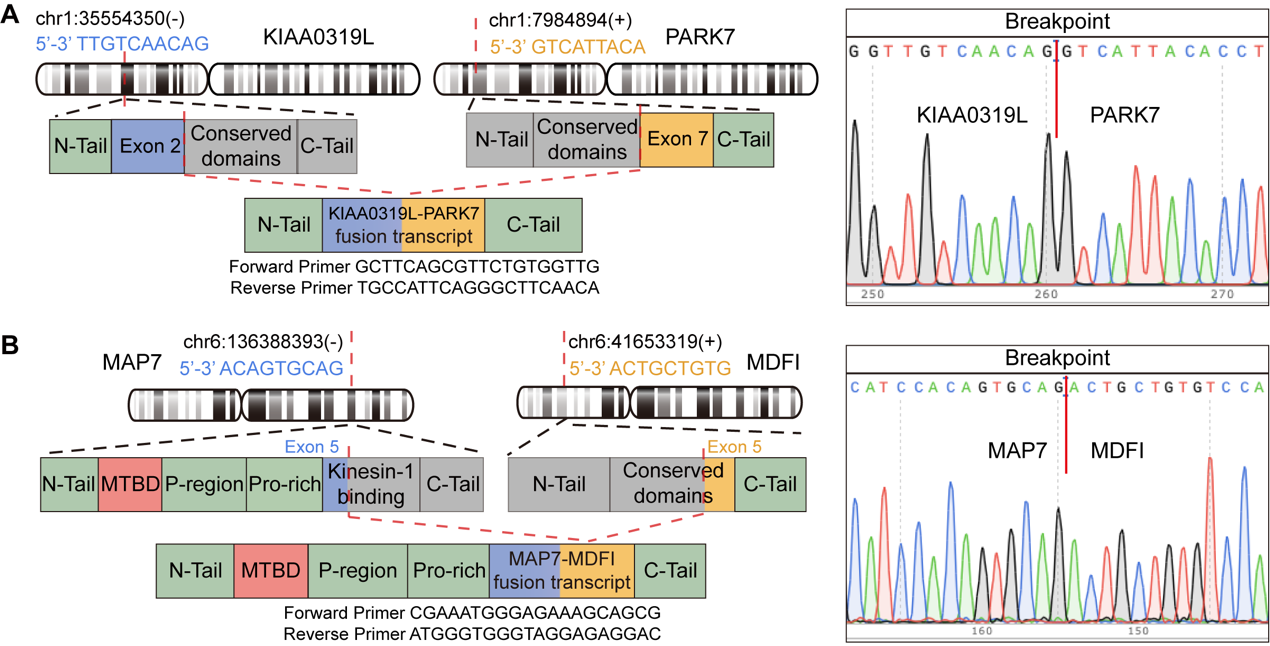
**Supplementary Figure 4.** **Schematic and Sanger sequencing of fusion genes**

(A), Fusion gene *KIAA0319L-PARK7* was identified in SP-EPN 6. (B), Fusion gene *MAP7-MDFI* was identified in SP-SE 1. Left panel: Schematic representations of the *KIAA0319L-PARK7* and *MAP7-MDFI* fusion gene structures. Right panel: Sanger sequencing chromatograms validating the fusion breakpoints. The red vertical line indicates the precise breakpoint position.


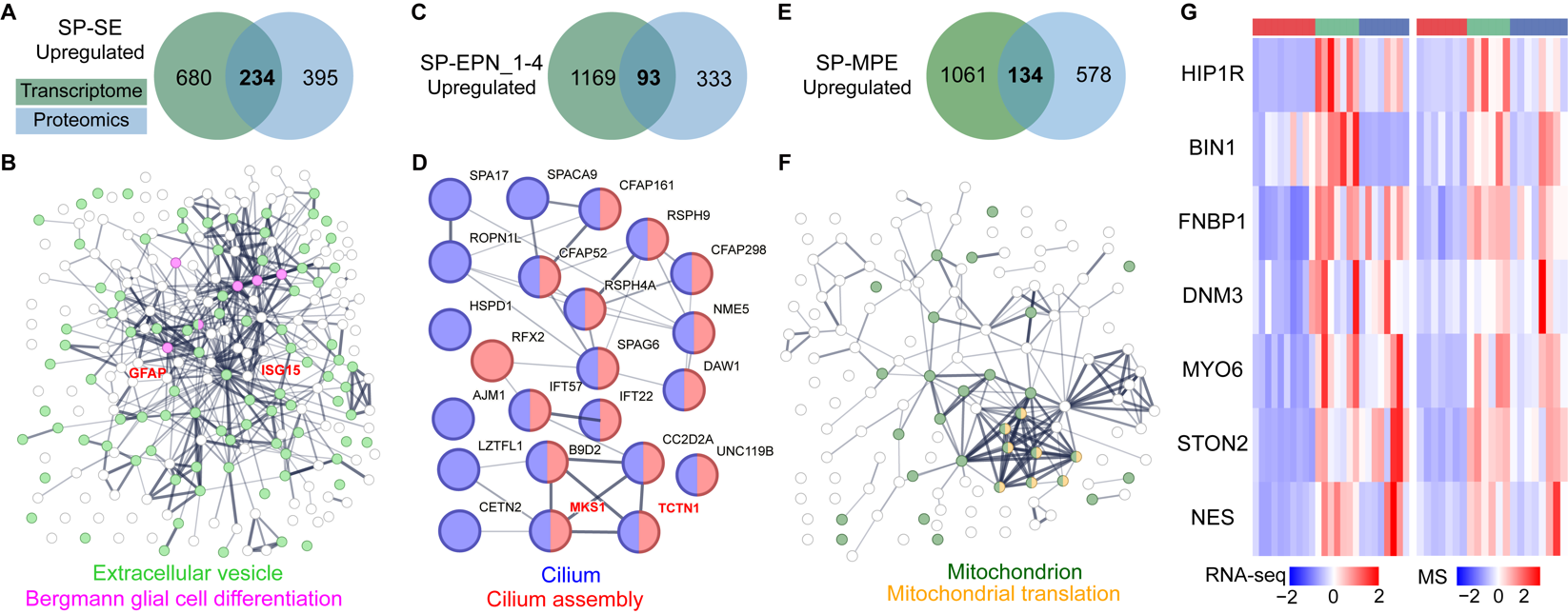
**Supplementary Figure 5. Integrative Analysis of Spinal Ependymal Tumors**

**(A, C, E),** The Venn diagram depicts the overlapping upregulated genes in both transcriptome and proteome of SP-SE, grade 2 SP-EPN_1-4, SP-MPE. **(B, D, F),** Protein-protein interaction network of the overlapping genes in the three subtypes. **(G),** Heatmaps of down-regulated TCA-related genes in SP-MPE, based on RNA data (left) and protein data (right). PPI network centrality: Betweenness centrality calculated via STRING package^38^.
