# Supplementary material for: Multi-omics analysis delineates molecular signatures of spinal ependymal tumor
Source: Cell Oncol (Dordr). 2025 Oct 29;48(6):1987–2000. doi: 10.1007/s13402-025-01122-0 (PMC12698791; doi:10.1007/s13402-025-01122-0)
Supplement: Supplementary file 2 — Supplementary Material 2 [file 13402_2025_1122_MOESM2_ESM.docx]

Table S1. Clinical characteristics of the study cohort (n = 25).

| Age at diagnosis | 39.8 y (mean);  13–67 y (range) |
| --- | --- |
| Gender |  |
| Female | 10 |
| Male | 15 |
| Location |  |
| Cervical | 12 |
| Thoracic | 3 |
| Lumbosacrum | 10 |
| Histologic grade* |  |
| 1 | 7 |
| 2 | 14 |
| 3 | 4 |
| MYCN amplification | 2 |
| NTRK2 amplification | 5 |
| NTRK3 amplification | 6 |
| KRAS amplification | 4 |
| NOTCH1 amplification | 4 |
| Surgery |  |
| Gross-total resection | 20 |
| Subtotal resection | 5 |

*Graded according to WHO 2021 CNS5 Guidelines.
