# Supplementary material for: Multi-omics analysis delineates molecular signatures of spinal ependymal tumor
Source: Cell Oncol (Dordr). 2025 Oct 29;48(6):1987–2000. doi: 10.1007/s13402-025-01122-0 (PMC12698791; doi:10.1007/s13402-025-01122-0)
Supplement: Supplementary file 3 — Supplementary Material 3 [file 13402_2025_1122_MOESM3_ESM.docx]

Supplementary Table S2. Clinicopathologic information of 25 spinal ependymal tumor patients

| Patient ID | Gender | Age | Location | Resection | Tumor type | *MYCN* copy number | | | *MYCN* staining | PFS  (month) |
| --- | --- | --- | --- | --- | --- | --- | --- | --- | --- | --- |
|  |  |  |  |  |  | WGS | | Panel seq |  |  |
| SP-EPN_1 | F | 28 | C1-4 | GTR | SP-EPN | 2.0 | | 2.0 | Neg | 20 |
| SP-EPN_2 | F | 37 | C1-7 | GTR | SP-EPN | 2.0 | | 2.0 | Neg | 19 |
| SP-EPN_3 | F | 31 | C3-5 | GTR | SP-EPN | 3.0 | | 2.7 | Neg | 24 |
| SP-EPN_4 | M | 65 | C3-T2 | GTR | SP-EPN | 2.9 | | 2.8 | Neg | 22 |
| SP-EPN_5 | F | 44 | C2-T3 | GTR | SP-EPN | 2.0 | | - | Neg | 35 |
| SP-EPN_6 | M | 34 | C3-6 | STR | SP-EPN | 2.0 | | - | Neg | 11 |
| SP-EPN_7 | M | 36 | C4-6 | STR | SP-EPN | 2.1 | | - | Neg | 35 |
| SP-EPN_8 | M | 43 | L2-S2 | STR | SP-EPN | 1.8 | | 1.6 | Neg | 12 |
| SP-SE_1 | M | 54 | T8-12 | GTR | SP-SE | 2.1 | - | | - | 16 |
| SP-SE_2 | M | 22 | C1-6 | GTR | SP-SE | 2.0 | - | | - | 18 |
| SP-SE_3 | M | 48 | T3-8 | GTR | SP-SE | 2.1 | - | | - | 43 |
| SP-SE_4 | M | 48 | C3-6 | GTR | SP-SE | 1.9 | - | | - | 35 |
| SP-SE_5 | F | 67 | C2-6 | STR | SP-SE | 2.1 | - | | - | 46 |
| SP-SE_6 | M | 37 | C6-T4 | GTR | SP-SE | 1.8 | - | | - | 33 |
| SP-SE_7 | M | 24 | C2-C7 | GTR | SP-SE | 1.8 | - | | - | 21 |
| SP-MPE_1 | F | 28 | T10-S2 | GTR | SP-MPE | 2.0 | - | | - | 70 |
| SP-MPE_2 | M | 44 | L2-3, S1-2 | GTR | SP-MPE | 2.1 | 2.1 | | - | 9 |
| SP-MPE_3 | M | 43 | L1-S5 | GTR | SP-MPE | 1.9 | 1.7 | | - | 22 |
| SP-MPE_4 | M | 13 | L4-S1 | GTR | SP-MPE | 1.9 | 1.9 | | - | 33 |
| SP-MPE_5 | F | 52 | L2-3 | GTR | SP-MPE | 1.7 | - | | - | 27 |
| SP-MPE_6 | M | 34 | L3-4 | GTR | SP-MPE | 2.1 | - | | - | 33 |
| SP-MPE_7 | F | 35 | L1-3 | GTR | SP-MPE | 2.0 | - | | - | 41 |
| SP-MPE_8 | F | 39 | Lumbosacral multiple | STR | SP-MPE | 2.1 | - | | - | 10 |
| SP-MPE_9 | M | 32 | S3-4 | GTR | SP-MPE | 1.8 | - | | - | 18 |
| SP-MPE_10 | F | 57 | L1-2 | GTR | SP-MPE | 2.0 | - | | - | 27 |

GTR, Gross total resection; STR, Subtotal resection. PFS, Progression-free survival;
