# Supplementary material for: Multi-omics analysis delineates molecular signatures of spinal ependymal tumor
Source: Cell Oncol (Dordr). 2025 Oct 29;48(6):1987–2000. doi: 10.1007/s13402-025-01122-0 (PMC12698791; doi:10.1007/s13402-025-01122-0)
Supplement: Supplementary file 4 — Supplementary Material 4 [file 13402_2025_1122_MOESM4_ESM.docx]

| Patient ID | GFAP | EMA | H3K27me3 | Olig-2 | S100 | SOX10 | Ki-67 |
| --- | --- | --- | --- | --- | --- | --- | --- |
| SP-EPN_1 | **+** | **+** | **+** | **-** | **/** | **/** | 1-2% |
| SP-EPN_2 | **+** | **+** | **+** | **-** | **/** | **/** | 1% |
| SP-EPN_3 | **+** | **-** | **+** | **/** | **/** | **/** | 3% |
| SP-EPN_4 | **+** | **/** | **+** | **/** | **/** | **/** | <5% |
| SP-EPN_5 | **+** | **/** | **/** | **/** | **/** | **/** | <5%, part 5-10% |
| SP-EPN_6 | **+** | **-** | **/** | **+** | **/** | **/** | 10-15% |
| SP-EPN_7 | **+** | **-** | **+** | **+** | **/** | **/** | 15% |
| SP-EPN_8 | **+** | **+** | **+** | **/** | **/** | **/** | 10-15% |
| SP-SE_1 | **+** | **-** | **+** | **/** | **/** | **/** | <1% |
| SP-SE_2 | **/** | **/** | **/** | **/** | **/** | **/** | <5% |
| SP-SE_3 | **/** | **/** | **/** | **/** | **/** | **/** | <1% |
| SP-SE_4 | **+** | **-** | **/** | **+** | **/** | **/** | 2% |
| SP-SE_5 | **+** | **+** | **/** | **-** | **/** | **-** | 1% |
| SP-SE_6 | **+** | **-** | **/** | **-** | **/** | **/** | 1-3%, part 10% |
| SP-SE_7 | **+** | **+** | **+** | **/** | **/** | **/** | 3-15% |
| SP-MPE_1 | **+** | **-** | **+** | **-** | **/** | **/** | 1-2% |
| SP-MPE_2 | **+** | **+** | **-** | **+** | **/** | **/** | 1% |
| SP-MPE_3 | **+** | **/** | **/** | **/** | **/** | **/** | <3% |
| SP-MPE_4 | **/** | **+** | **+** | **/** | **/** | **/** | 1-5% |
| SP-MPE_5 | **+** | **-** | **+** | **-** | **/** | **/** | 1-3% |
| SP-MPE_6 | **+** | **-** | **+** | **-** | **-** | **-** | **+** |
| SP-MPE_7 | **/** | **-** | **+** | **/** | **/** | **/** | 3-7% |
| SP-MPE_8 | **+** | **+** | **+** | **-** | **/** | **/** | 1-2%, part 15% |
| SP-MPE_9 | **+** | **-** | **/** | **+** | **+** | **/** | Part 10% |
| SP-MPE_10 | **+** | **+** | **+** | **+** | **/** | **/** | 1-3%, part 10% |

**Table S3. Immunohistochemical results of 25 cases of spinal ependymal tumors**

+, Positive; -, Negative; /, Not tested.
