# Supplementary material for: Multi-omics analysis delineates molecular signatures of spinal ependymal tumor
Source: Cell Oncol (Dordr). 2025 Oct 29;48(6):1987–2000. doi: 10.1007/s13402-025-01122-0 (PMC12698791; doi:10.1007/s13402-025-01122-0)
Supplement: Supplementary file 5 — Supplementary Material 5 [file 13402_2025_1122_MOESM5_ESM.zip › μû░σó₧ΘÖäΣ╗╢/Methods for SNP and CNV Analysis.docx]

Variant Calling and Annotation

To ensure high-quality variant calling, we first performed read quality control using fastp (Chen et al., *Bioinformatics*, 2018) with the following parameters:
fastp -i read_1.fq.gz -o read_1.fp.fastq.gz -I read_2.fq.gz -O read_2.fp.fastq.gz -z 4 -f 5 -t 5 -F 5 -T 5 -5 -W 5 -M 20 -Q -l 50 -c -w 4.

Next, we further trimmed adapter sequences and low-quality bases using

Trimmomatic v0.39(Bolger *et al.* Bioinformatics. 2014) with the command:
java -Xmx8G -jar trimmomatic-0.39.jar PE -threads 6 read_1.fp.fastq.gz read_2.fp.fastq.gz read_1P.fq.gz read_1U.fq.gz read_2P.fq.gz read_2U.fq.gz ILLUMINACLIP:TruSeq3-PE.fa:2:30:10 MINLEN:36 HEADCROP:3 2>clip.log.

Cleaned paired-end reads were aligned to the human reference genome (hg38) using BWA-MEM(Li *et al.* Bioinformatics. 2009) with the following parameters:
bwa mem -t 4 -R '@RG\tID:foo_lane\tPL:illumina\tLB:library\tSM:sample_name' human.fasta read_1.fq.gz read_2.fq.gz | samtools view -S -b - > sample_name.bam.

To ensure high confidence in downstream variant calling, we performed base quality score recalibration (BQSR) using GATK(McKenna *et al.* Genome Res. 2010). First, we sorted the BAM file using Picard:
java -jar picard.jar ReorderSam I=dedupped.bam O=ReSort.bam REFERENCE=hg38.fa
and indexed it with:
java -jar picard.jar BuildBamIndex I=ReSort.bam.

Local realignment around indels was performed using known gold-standard indel sites:

java -jar GenomeAnalysisTK.jar -T RealignerTargetCreator -R hg38.fa -I ReSort.bam -o target_intervals.list -known indel/Mills_and_1000G_gold_standard.indels.hg38.vcf.gz

java -jar GenomeAnalysisTK.jar -T IndelRealigner -R hg38.fa -I ReSort.bam -o realigned.bam -targetIntervals target_intervals.list -known indel/Mills_and_1000G_gold_standard.indels.hg38.vcf.gz

java -jar GenomeAnalysisTK.jar -T RealignerTargetCreator -R hg38.fa -I ReSort.bam -o target_intervals.list -known indel/Mills_and_1000G_gold_standard.indels.hg38.vcf.gz

java -jar GenomeAnalysisTK.jar -T IndelRealigner -R hg38.fa -I ReSort.bam -o realigned.bam -targetIntervals target_intervals.list -known indel/Mills_and_1000G_gold_standard.indels.hg38.vcf.gz

Base quality scores were recalibrated using:

java -jar GenomeAnalysisTK.jar -T BaseRecalibrator -R hg38.fa -I realigned.bam -o recal.table -knownSites hg38_v0_Homo_sapiens_assembly38.dbsnp138.vcf.gz -knownSites Mills_and_1000G_gold_standard.indels.hg38.vcf.gz

java -jar GenomeAnalysisTK.jar -T PrintReads -R hg38.fa -I realigned.bam -o final.bqsr.bam -BQSR recal.table

Joint genotyping was performed on GVCFs using:
java -jar GenomeAnalysisTK.jar -T GenotypeGVCFs -R hg38.fa -maxAltAlleles 20 -stand_call_conf 20.0 --variant all_merge.rawgvcf -o all.HC.vcf.

Variant quality score recalibration (VQSR) was applied in two stages:

For SNPs:

java -jar GenomeAnalysisTK.jar -T VariantRecalibrator -R hg38.fa -input all.HC.vcf \

-resource:hapmap,known=false,training=true,truth=true,prior=15.0 hapmap_3.3.hg38.vcf.gz \

-resource:omni,known=false,training=true,truth=false,prior=12.0 1000G_omni2.5.hg38.vcf.gz \

-resource:1000G,known=false,training=true,truth=false,prior=10.0 1000G_phase1.snps.high_confidence.hg38.vcf.gz \

-resource:dbsnp,known=true,training=false,truth=false,prior=6.0 hg38_v0_Homo_sapiens_assembly38.dbsnp138.vcf.gz \

-an QD -an MQ -an MQRankSum -an ReadPosRankSum -an FS -an SOR -an DP \

-mode SNP -recalFile snps.recal -tranchesFile snps.tranches -rscriptFile snps.plots.R

java -jar GenomeAnalysisTK.jar -T ApplyRecalibration -R hg38.fa -input all.HC.vcf \

--ts_filter_level 99.5 -tranchesFile snps.tranches -recalFile snps.recal -mode SNP \

-o filtered.snps.vcf

For INDELs:

java -jar GenomeAnalysisTK.jar -T VariantRecalibrator -R hg38.fa -input filtered.snps.vcf \

-resource:mills,known=true,training=true,truth=true,prior=12.0 Mills_and_1000G_gold_standard.indels.hg38.vcf.gz \

-an QD -an DP -an FS -an SOR -an ReadPosRankSum -an MQRankSum \

-mode INDEL -recalFile indels.recal -tranchesFile indels.tranches -rscriptFile indels.plots.R

Further hard filtering of variants was applied using:

java -jar GenomeAnalysisTK.jar -T VariantFiltration -R hg38.fa -V all.HC.vcf -o final.vcf \

-window 35 -cluster 3 -filterName FS -filter "FS > 30" -filterName QD -filter "QD < 2"

SNPs and indels were then extracted separately:

java -jar GenomeAnalysisTK.jar -T SelectVariants -R hg38.fa -V final.vcf -o final.indel.vcf -selectType INDEL

java -jar GenomeAnalysisTK.jar -T SelectVariants -R hg38.fa -V final.vcf -o final.SNP.vcf -selectType SNP

Finally, variant annotation was conducted using SnpEff:

java -jar snpEff.jar download GRCh38.76

java -jar snpEff.jar hg38 -v final.SNP.vcf -c snpEff.config -o gatk > final.snp.anno.vcf

CNV call by cnvkit with parameters:

cnvkit.py batch sample.sorted.markdup.relign.BQSR.bam -n --output-reference my_reference.cnn --output-dir cnvkit -p 6 --method wgs --drop-low-coverage --scatter --diagram --fasta hg38.fa --annotate hg38.refFlat.txt

Bolger, Anthony M., Marc Lohse & Bjoern Usadel. Trimmomatic: a flexible trimmer for Illumina sequence data. *Bioinformatics* (2014)*,* 30, 2114-2120.

Li, Heng & Richard Durbin. Fast and accurate short read alignment with Burrows–Wheeler transform. *Bioinformatics* (2009)*,* 25, 1754-1760.

McKenna, A., M. Hanna, E. Banks, A. Sivachenko, K. Cibulskis*, et al.* The Genome Analysis Toolkit: a MapReduce framework for analyzing next-generation DNA sequencing data. *Genome Res* (2010)*,* 20, 1297-303.
