# Supplementary material for: Multi-omics analysis delineates molecular signatures of spinal ependymal tumor
Source: Cell Oncol (Dordr). 2025 Oct 29;48(6):1987–2000. doi: 10.1007/s13402-025-01122-0 (PMC12698791; doi:10.1007/s13402-025-01122-0)
Supplement: Supplementary file 5 — Supplementary Material 5 [file 13402_2025_1122_MOESM5_ESM.zip › μû░σó₧ΘÖäΣ╗╢/Targeted gene panel sequencing/SGM2 181709-1-report.pdf]

基本信息及检测项目

1 基本信息

病理编号	181709-1	报告日期	2022-07-09

2 检测项目

检测项目：儿童肿瘤精准诊疗基因检测

检测方法：二代测序法

检测内容：

本项目共检测 86个基因的点突变、插入、缺失：

ABL1	ABL2	ALK	ACVR1	AKT1	ASXL1	ASXL2	BRAF	CALR	CBL	CCND1	CCND3
CCR5	CDK4	CIC	CREBBP	CRLF2	CSF1R	CSF3R	CTNNB1	DAXX	DNMT3A	EGFR	EP300
ERBB2	ERB83	ERBB4	ESR1	EZH2	FASLG	FBXW7	FGFR1	FGFR2	FGFR3	FLT3	GATA2
GNA11	GNAQ	H3F3A	HDAC9	HIST1H3B	HRAS	IDH1	IDH2	IL7R	JAK1	JAK2	JAK3
KDM4C	KDR	KIT	KRAS	MAP2K1	MAP2K2	MET	MPL	MSH6	MTOR	MYC	MYCN
NCOR2	NOTCH 1	NPM1	NRAS	NT5C2	PAX5	PDGFRA	PDGFRB	PIK3CA	P1K3R1	PPM1D	PTPN11
RAFI	RET	RHOA	SETBP1	SETD2	SH2B3	SH2D1A	SMO	STAT3	STAT5B	TERT	TPMT
USP7	ZMYM3										

本项目共检测 28个基因的拷贝数变异：

ABL2	ALK	BRAF	CCND1	CDK4	CDK6	EGFR	ERB82	ERB83	FGFR1	FGFR2	FGFR3
FGFR4	GLI1	GLI2	IGF1R	JAK1	JAK2	JAK3	KIT	KRAS	MDM2	MDM4	MET
MYC	MYCN	PDGFRA	PIK3CA								

本项目共检测 44个基因全外显子：

APC	ARID1A	ARID1B	ATRX	COKN2A	CDKN2B	CEBPA	CHD7	CRLF1	DDX3X	DICER1	EBF1
EED	FAS	GATA1	GATA3	GNA13	ID3	IKZF1	KDM6A	KMT2D	MYOD1	NF1	NF2
PHF6	PRPS1	PSMB5	PTCH1	PTEN	RB1	RUNX1	SMARCA4	SMARCB1	SOCS2	SUFU	SUZ12
TCF3	TET2	TP53	TSC1	TSC2	WHSC1	WT1	XIAP				

本项目共检测 88个基因重排（融合）：

ABL1	ABL2	AFF3	ALK	BCL11B	BCOR	BCR	BRAF	CAMTA1	CCND1	CIC	CREBBP
CRLF2	CSF1R	DUSP22	EGFR	ETV6	EWSR1	FGFR1	FGFR2	FGFR3	FLT3	FOSB	FUS
GLI1	GLIS2	HMGA2	JAK2	KAT6A	KMT2A	KMT2B	KMT2C	KMT2D	LMO2	MAML2	MAN2B1
MECOM	MEF2D	MET	MKL1	MLLT10	MN1	MYB	MYBL1	MYH11	MYH9	NCOA2	NCOR1
NOTCH1	NOTCH2	NOTCH4	NPM1	NR4A3	NTRK1	NTRK2	NTRK3	NUP214	NUP98	NUTM1	NUTM2B
PAX3	PAX5	PAX7	PDGFB	PDGFRA	PDGFRB	PLAG1	RAF1	RANBP17	RARA	RECK	RELA
RET	ROS1	RUNX1	SS18	SSBP2	STAG2	STAT6	TAL1	TCF3	TFE3	TP63	TSLP
TSPAN4	UBTF	USP6	WHSC1	YAP1	ZMYND11	ZNF384					

检测意义：1.预测诊断分型 2.预测临床预后 3.预测药物有效性

检测结果及临床分析

1 检测项目结果小结

检测内容	检测结果
基因位点突变、插入、缺失	GNAQ: p.Gln209Lys;
拷贝数分析	未见拷贝数变异
基因重排（融合）分析	未见基因重排

2 基因变异分析结果

2.1 基因位点突变、插入、缺失分析结果

基因	染色体	转录本号	外显子	核苷酸变化	氨基酸变化	突变类型	变异丰度(%)
GNAQ	9	NM_002072.4	exon5	c.625C>A	p.Gln209Lys	错义突变	4.13

2.2 拷贝数分析结果

基因/染色体区段	染色体	变异类型	拷贝数
无	无	无	无

2.3 基因重排分析结果

重排基因	断点位置	外显子	Reads数
无	无	无	无

注：

1.根据人类基因组突变学会（HGVS）已建立系统的基因突变命名方法，“c.”代表 cDNA 序列，“p.”代 表蛋白质序列。

2.在 DNA 水平，对某一突变位点的描述方式包括碱基位点，正常碱基，“>”符号，突变碱基。

3.在氨基酸水平，其表示方法是野生型的氨基酸，位点，突变氨基酸，三者之间没有空格。

4.变异丰度：肿瘤组织检测数据中，支持该基因位点变异的分子数占该位点总分子数的比例。因测序深度和肿瘤取样部位不同，变异丰度可能会存在一定差异。
