# Supplementary figures and images for: Multi-omics analysis delineates molecular signatures of spinal ependymal tumor
Source: Cell Oncol (Dordr). 2025 Oct 29;48(6):1987–2000. doi: 10.1007/s13402-025-01122-0 (PMC12698791; doi:10.1007/s13402-025-01122-0)

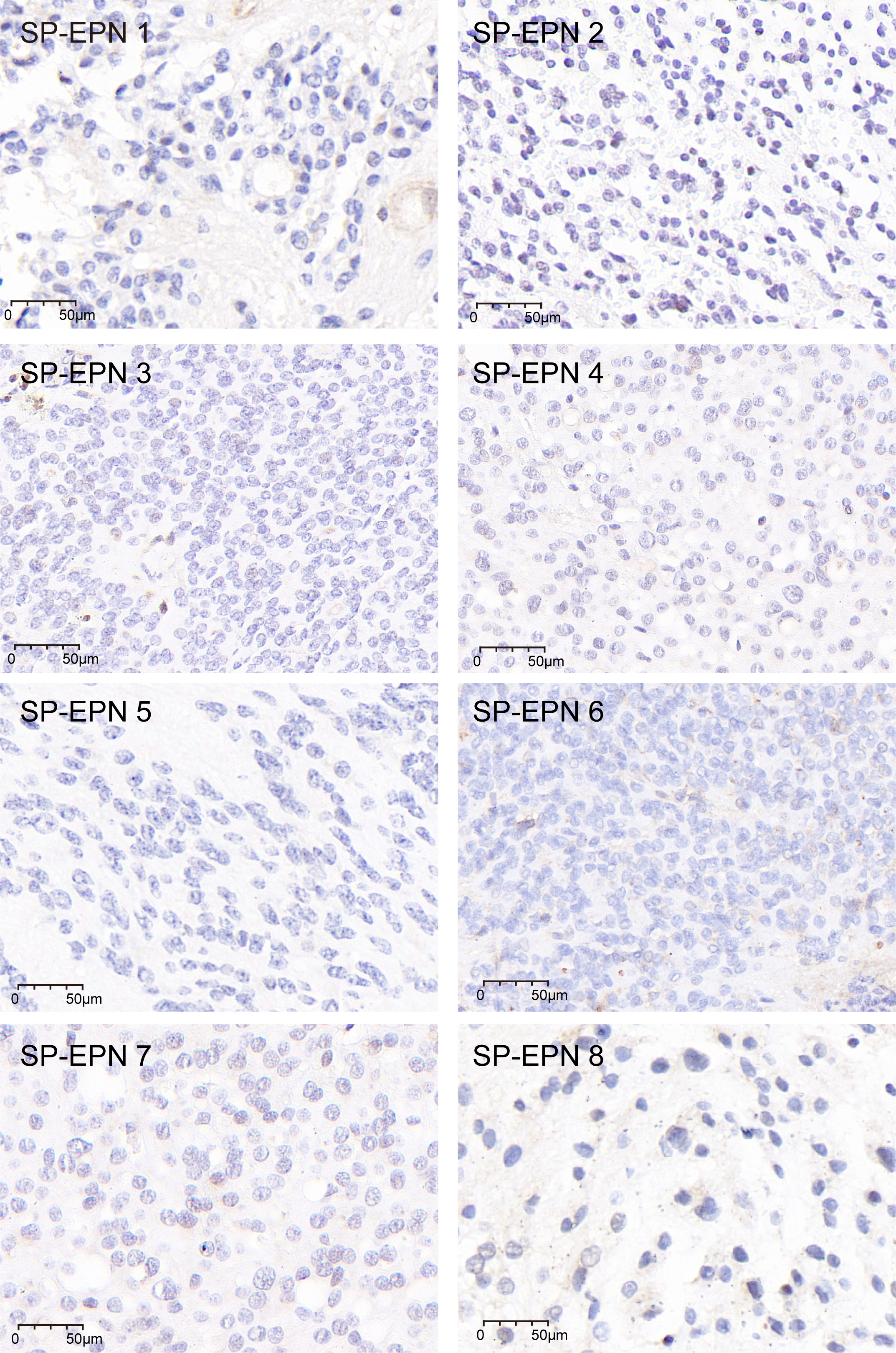

Supplement: Supplementary file 7 — Supplementary Material 7 [file 13402_2025_1122_MOESM7_ESM.png]

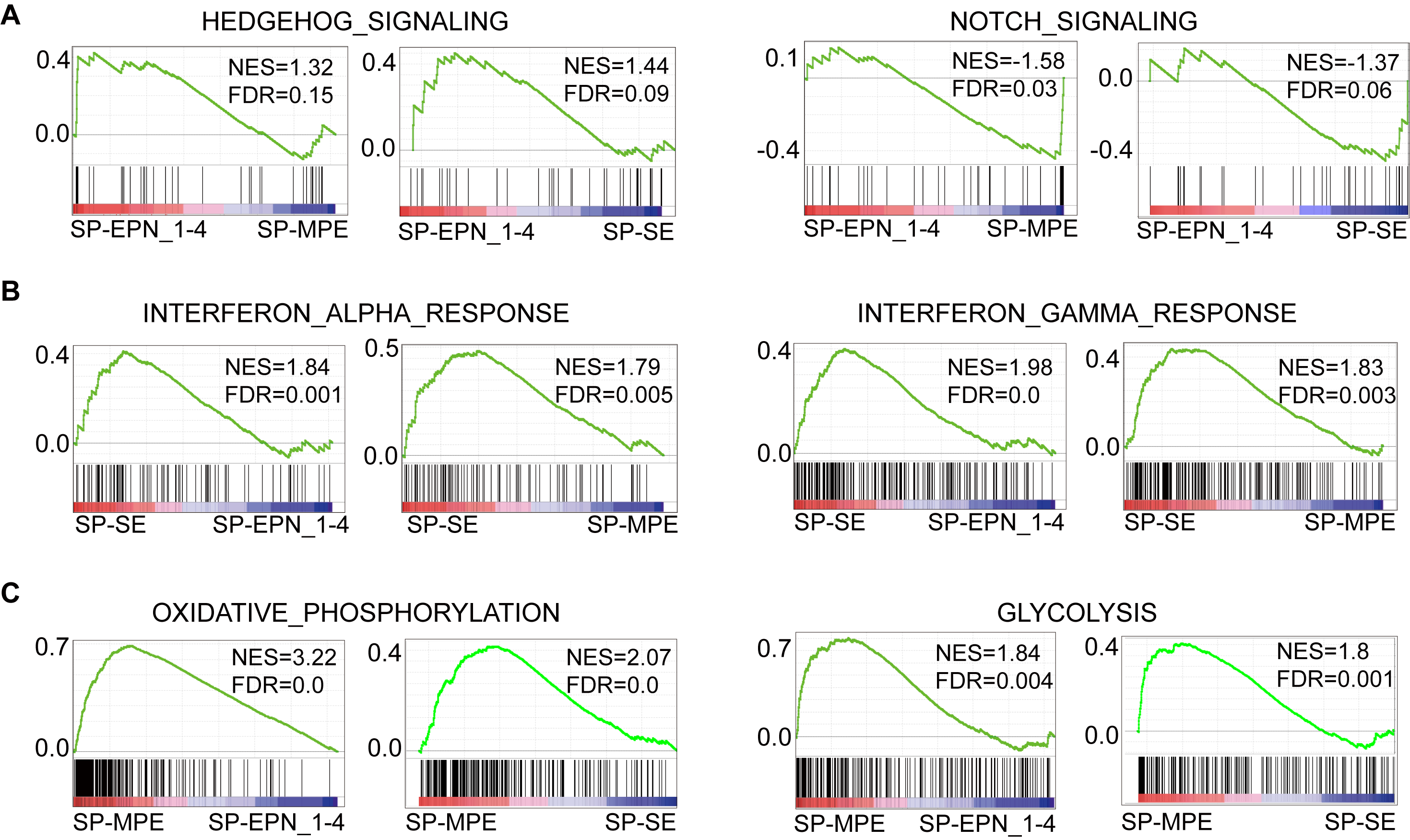

Supplement: Supplementary file 8 — Supplementary Material 8 [file 13402_2025_1122_MOESM8_ESM.png]

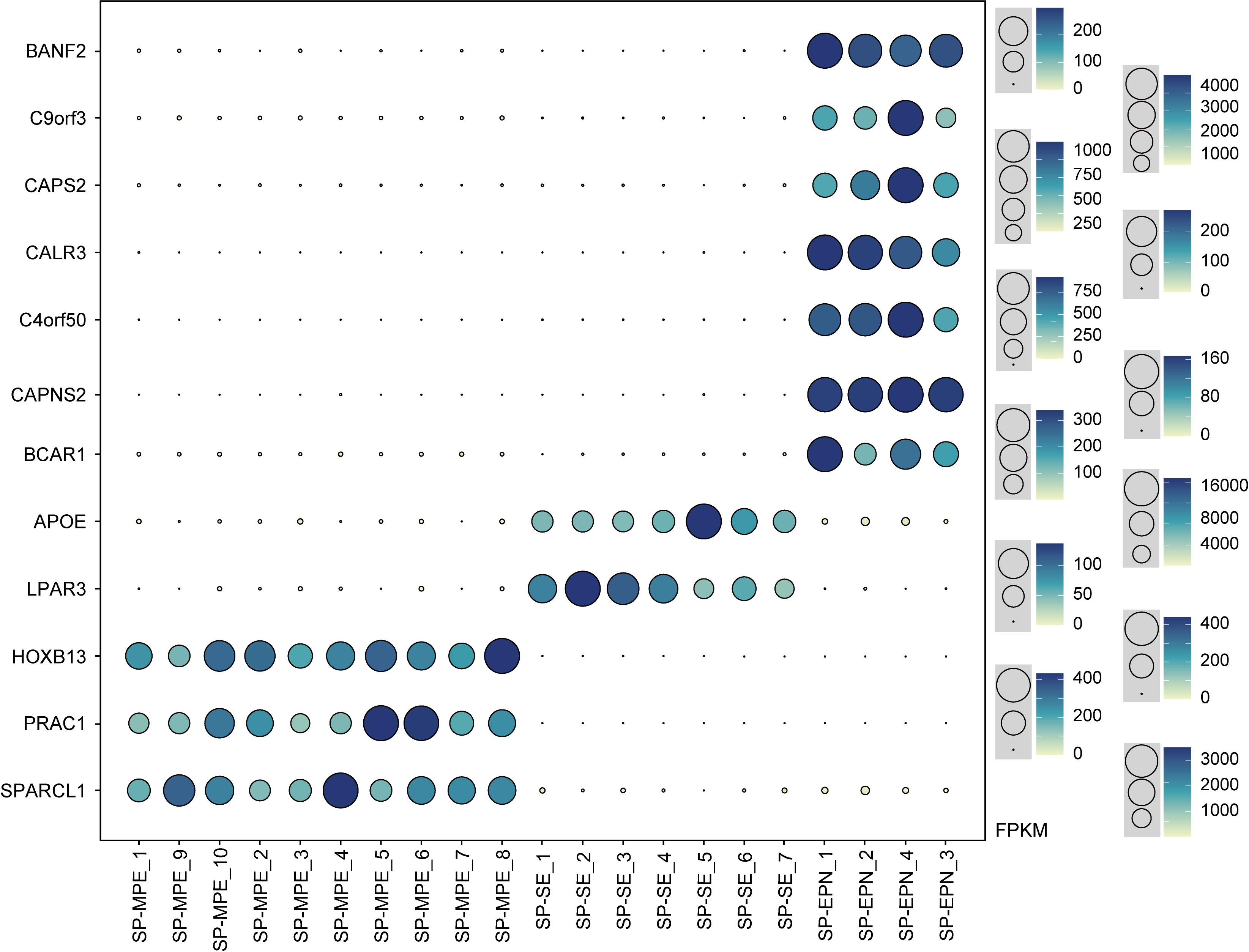

Supplement: Supplementary file 9 — Supplementary Material 9 [file 13402_2025_1122_MOESM9_ESM.png]

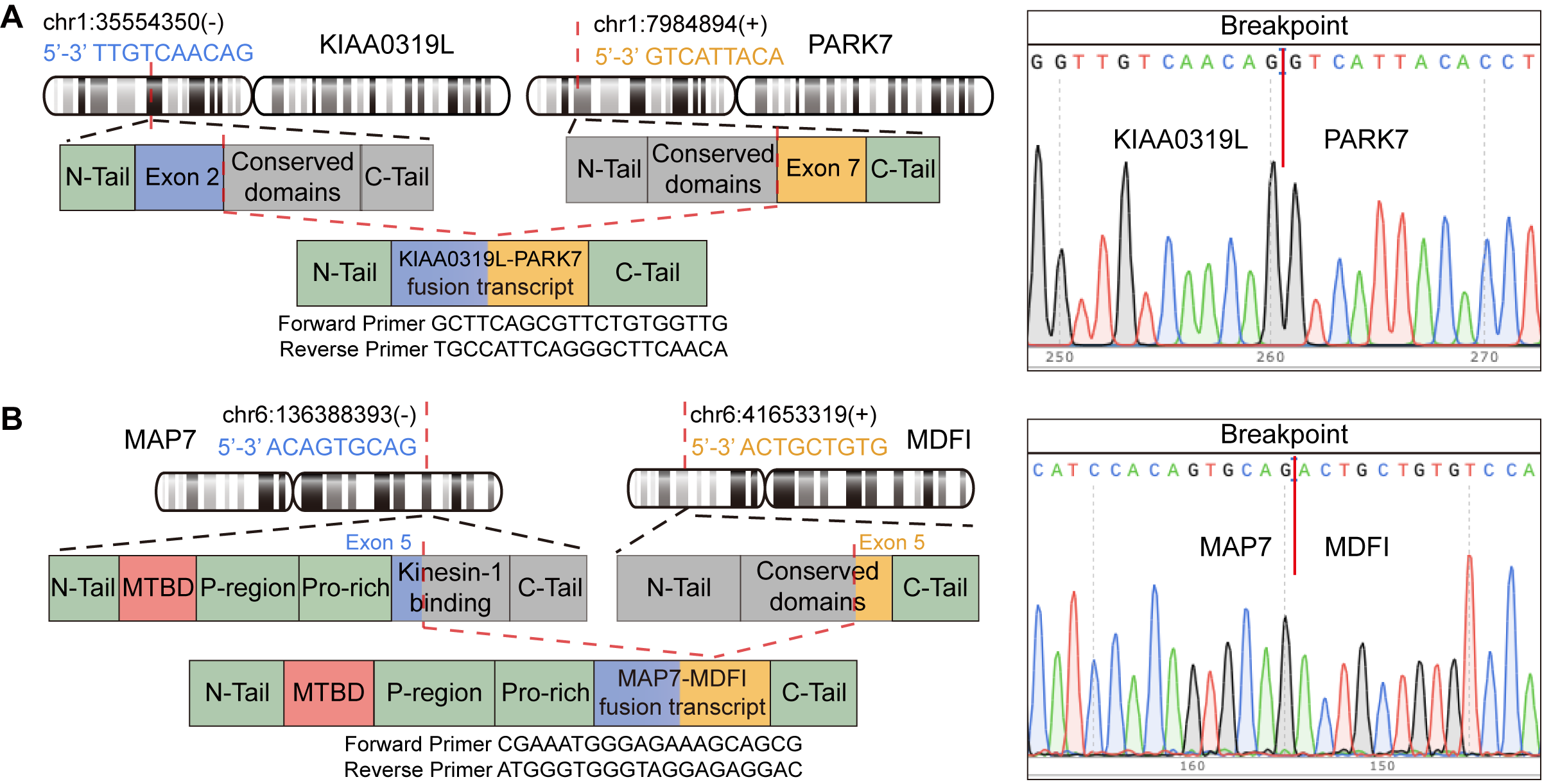

Supplement: Supplementary file 10 — Supplementary Material 10 [file 13402_2025_1122_MOESM10_ESM.png]

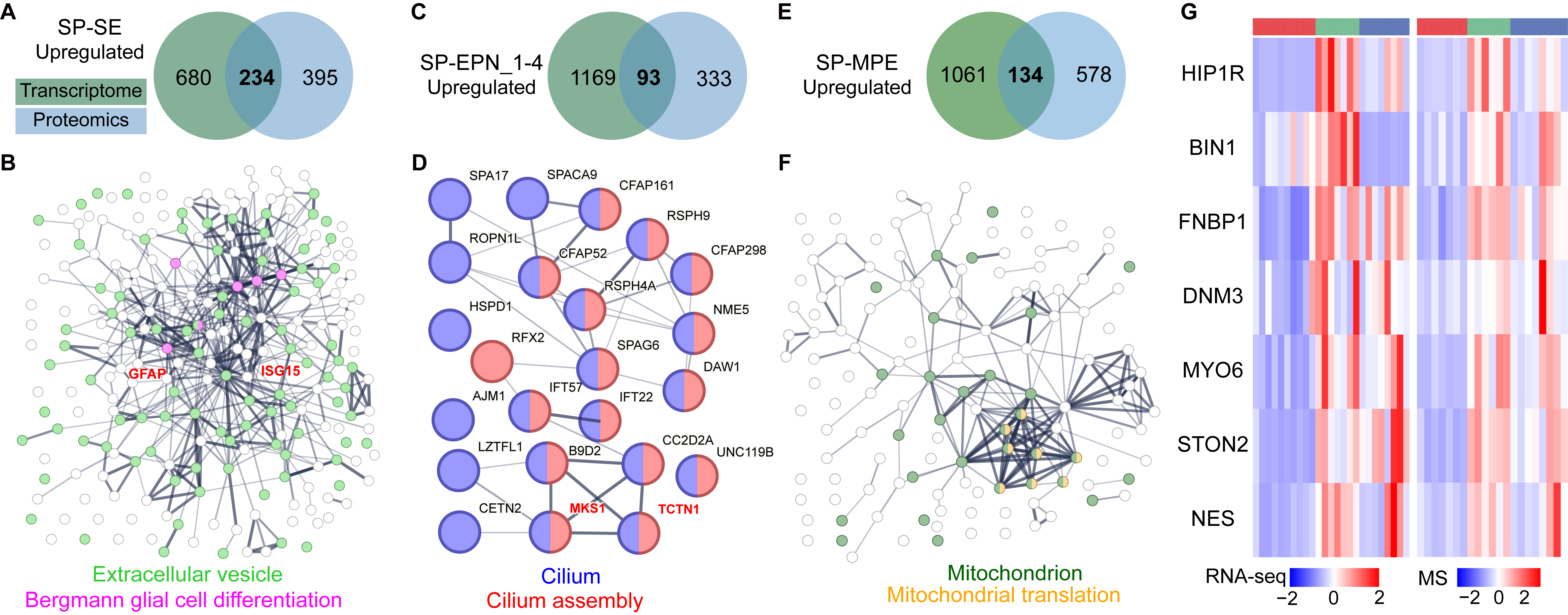

Supplement: Supplementary file 11 — Supplementary Material 11 [file 13402_2025_1122_MOESM11_ESM.png]
